# A miR-SNP biomarker linked to an increased lung cancer survival by miRNA-mediated down-regulation of FZD4 expression and Wnt signaling

**DOI:** 10.1038/s41598-017-09604-4

**Published:** 2017-08-22

**Authors:** Jing Lin, Roza Zandi, Ruping Shao, Jian Gu, Yuanqin Ye, Jing Wang, Yang Zhao, Alexander Pertsemlidis, Ignacio I. Wistuba, Xifeng Wu, Jack A. Roth, Lin Ji

**Affiliations:** 1 Section of Thoracic Molecular Oncology, Department of Thoracic and Cardiovascular Surgery, Houston, TX 77030 USA; 2 Department of Epidemiology, The University of Texas MD Anderson Cancer Center, Houston, TX 77030 USA; 3 Department of Bioinformatics & Computational Biology, The University of Texas MD Anderson Cancer Center, Houston, TX 77030 USA; 4 Department of Translational Molecular Pathology, The University of Texas MD Anderson Cancer Center, Houston, TX 77030 USA; 5 Greehey Children’s Cancer Research Institute, The University of Texas Health Science Center, San Antonio, TX 78229 USA

## Abstract

Through a new hypothesis-driven and microRNA-pathway-based SNP (miR-SNP) association study we identified a novel miR-SNP (rs713065) in the 3′UTR region of FZD4 gene linked with decreased risk of death in early stage NSCLC patients. We determined biological function and mechanism of action of this FZD4-miR-SNP biomarker in a cellular platform. Our data suggest that FZD4-miR-SNP loci may significantly influence overall survival in NSCLC patients by specifically interacting with miR-204 and modulating FZD4 expression and cellular function in the Wnt-signaling-driven tumor progression. Our findings are bridging the gap between the discovery of epidemiological SNP biomarkers and their biological function and will enable us to develop novel therapeutic strategies that specifically target epigenetic markers in the oncogenic Wnt/FZD signaling pathways in NSCLC.

## Introduction

Single nucleotide polymorphisms (SNPs) in miRNA genes and miRNA-associated pathways (miR-SNPs) have significant effects on gene expression and cellular processes by disrupting miRNA biogenesis and modulating miRNA-mRNA target interactions. A SNP (rs713065 with a C allele) biomarker in the 3′UTR of the FZD4 gene in wingless (Wnt) signaling pathways displayed a significant association with decreased risk of death in early stage NSCLC patients. We used a novel stem-loop array-reverse transcription-PCR (SLA-RT-PCR) assay to assess miRNA:target mRNA interaction at the specific FZD4-miR-SNP locus and detected miRNA-mediated FZD4 mRNA cleavage and 3′-uridylation in FZD4-SNP (C, rs713065) allele-bearing H1299 and H322 NSCLC cells, but not in FZD4-WT (T allele)-containing A549 and normal human bronchial epithelial cells. The presence of the FZD4-SNP in the 3′UTR down-regulated the ectopic expression of the host FZD4 gene and protein and inhibited tumor colony formation and tumor cell mobility in NSCLC cells. Furthermore, we identified miR-204 as a potential miRNA candidate that directly targets allelic variants at FZD4-miR-SNP loci. Target mRNA cleavage was detected at the SNP C allele site but not at the WT-allele T site in H1299 cells after co-transfection with a GFP-FZD4-SNP reporter and miR-204 expression plasmid constructs by SLA-RT-PCR. Multiple key genes in Wnt/FZD and Wnt-associated endothelial mesenchymal transition (EMT) signaling pathways were differentially modulated by the presence of FZD4-miR-SNP in NSCLC cells as demonstrated by a quantitative NanoString Wnt/EMT-pathway specific gene expression analysis. Our findings are bridging the gap between the epidemiological SNP biomarkers and their mechanisms of action in NSCLC.

Tumor-related germ line single nucleotide polymorphisms (SNPs) have been identified as potential prognostic and predictive markers for lung cancer outcomes and treatment response^1^. Genome-wide association studies (GWAS) have unequivocally identified many cancer susceptibility loci in SNPs^2^. However, the underlying biological mechanisms have been mostly unclear. The agnostic approach of GWAS without a biological hypothesis made the mechanistic study of identified genetic loci extremely challenging. Another more challenging question is that all of the GWAS-identified genetic variants had modest discriminatory ability and failed to significantly improve risk prediction. Individual SNP analysis without consideration of gene-gene and gene-environment interactions is unlikely to significantly improve risk prediction. To overcome these limitations of GWAS, a hypothesis-driven, pathway-based approach is a complementary, highly valuable strategy. By focusing on specific biological pathways that are closely related to disease pathogenesis, pathway-based approaches require smaller sample sizes, offer higher genomic coverage, and more reliably find gene-gene interactions and gene networks than *GWAS*. Many recent studies have demonstrated the value of the pathway-based approach in identifying novel genetic susceptibility SNPs for cancer risk^3–11^

MicroRNAs (miRNAs) are small (~22 nucleotides) noncoding RNAs that negatively regulate gene expression through complementary binding to the 3′ untranslated regions (3′UTRs) of their target messenger RNAs (mRNAs)^12^. miRNAs are incorporated into Argonaute^13^ proteins in RNA-induced silencing complexes (RISCs) leading to target mRNA cleavage and degradation^14^. SNPs in miRNA regulatory pathways (miR-SNPs) that could be included in three categories of genes: miRNA genes, miRNA biogenesis genes, and miRNA target genes, can affect the transcription and processing precursor miRNA (pre-miRNA), modulate the affinity of miRNA-mRNA binding, abolish an existing binding site, or create abnormal binding sites. These inherited genetic miR-SNP variants in miRNA binding sites within the 3′UTRs of target genes can significantly contribute to cancer risk and outcomes by regulating target gene expression and/or function^15, 16^. For example, a SNP in mature miR-196a2 was associated with poorer survival in NSCLC patients^17^. A pilot study showed that a haplotype in Drosha was significantly associated with shorter lung cancer survival and a SNP within the same haplotype was associated with reduced Drosha mRNA expression and resultant global miRNA expression changes in lung adenocarcinoma tissues^18^. The most exciting arena of miRNA SNPs is for those located within the miRNA binding sites of the 3′UTR of target genes^15, 16, 19, 20^. Chin *et al*.^21^ found an increased risk of lung cancer in subjects with a SNP in the 3′UTR of the KRAS gene that interferes with the binding of let-7 miRNA. A SNP in the 3′UTR of MDM4 caused the acquisition of an illegitimate miR-191 target site, reducing MDM4 expression, and significantly delaying ovarian carcinoma progression and tumor-related death^22^. Recently, a SNP in a target site for let-7 was identified in the *KRAS* 3′UTR and shown to be correlated with increased NSCLC risk^21^. Also, a SNP in the miR-125b binding site in the *BMPR1B* 3′UTRwas reported to disrupt miRNA repression of its target and confer increased risk of breast cancer^23^.

*FZD4* (frizzled homolog 4) is a transmembrane receptor that transduces Wnt signals, leading to nuclear accumulation of β–catenin and activation of WNT target genes^24^. Aberrant Wnt signaling underlies a wide range of pathologies in humans through the core canonical Wnt/beta-catenin signaling pathway leading to regulation of gene transcription and translation^25–27^. Dysregulation of Wnt-pathway-mediated cellular processes can cause unwanted cell growth and movement, which can lead to tumor development^28^ and is associated with tumor recurrence in patients with stage I NSCLC^29^. Recently, we found that the C allele of *FZD4*:rs713065 was linked with decreased risk of death in early stage NSCLC patients treated with surgery only^30^. In this study, we intend to identify endogenous miRNAs that are specifically interacting with Wnt-miR-SNPs and to determine how these Wnt-SNP- associated miRNAs differentially regulate their host/target gene expression and cellular function in NSCLC cells *in vitro* and *in vivo*. We will focus on to functionally characterize and validate the C allele of *FZD4-miR-SNP*, rs713065, in the miR-204 binding site in the *FZD4* 3′UTR, which was shown to be associated with a less aggressive NSCLC phenotype^30^ and to determine the biological and clinical relevance of this novel epidemiological FZD4-miR-SNP biomarker in NSCLC cell lines, preclinical mouse models, and in clinical plasma and tissue samples.

## Results

### Down-Regulated Expression of Reporter and FZD4 Genes by SNP variant rs713065(C)

**Figure 1:**
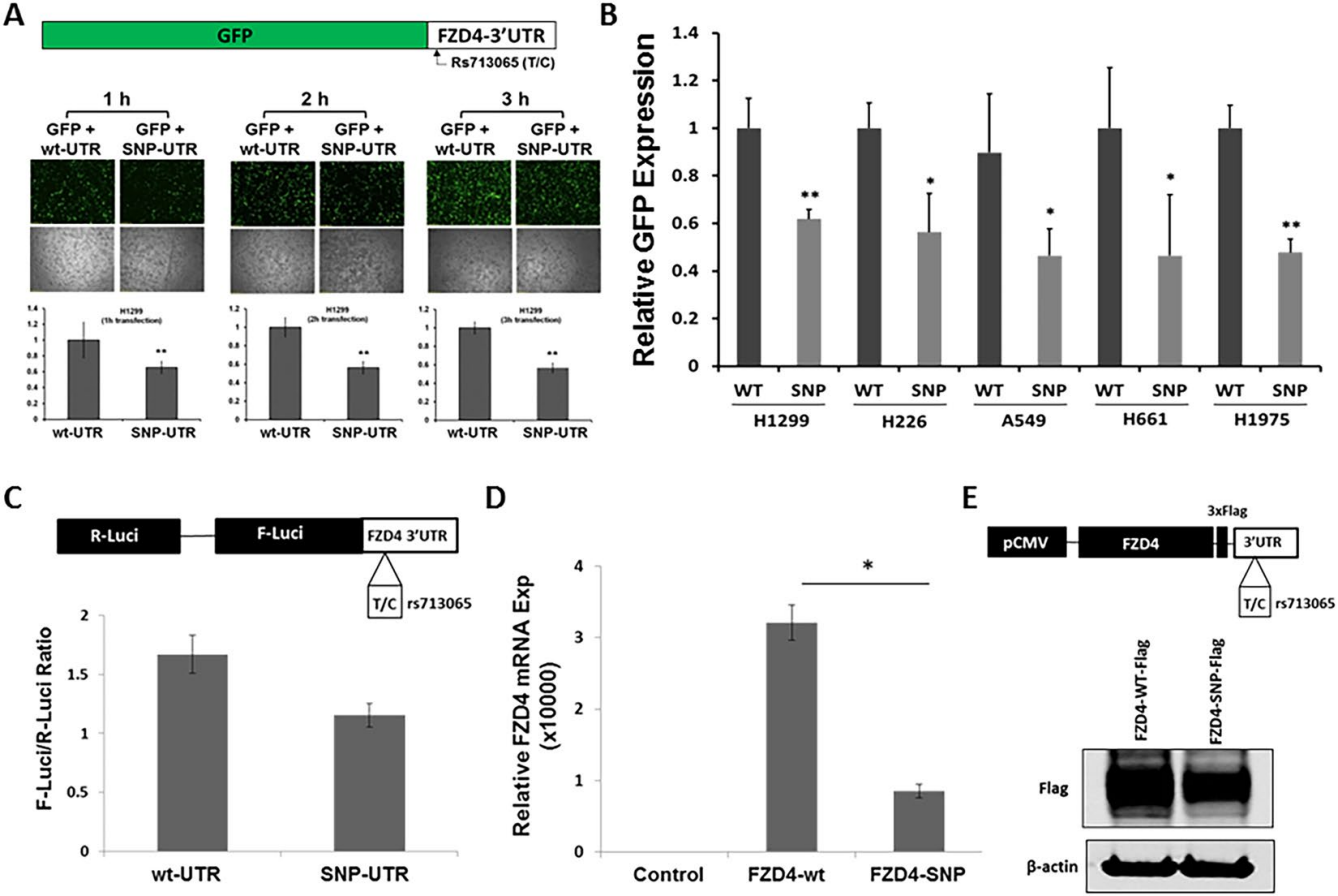
Effects of the variant allele rs713065(C) on FZD4 mRNA and protein expression. **(A**) The C allele significantly down-regulates expression of a reporter consisting of the coding sequence of GFP and part of the 3′UTR of FZD4 with variant (C) and wildtype (T) alleles at rs713065. Data are from six independent transfections. **(B)** Down-regulation of reporter gene expression by the rs713065(C) allele in H1299, H226, A549, H661, and H1975 cells. Data are from six independent transfections. **(C)** Dual luciferase reporter with the rs713065 variant allele. Firefly luciferase activity is normalized to sea pansy luciferase activity. The presence of the C allele results in the down-regulation of luciferase activity. Shown are the results of triplicate measurements (mean ± SE). **(D)** rs713065 (C) down-regulates FZD4 mRNA expression as determined by qRT-PCR (mean ± SE) (n = 3). *p < 0.05; **p < 0.001. (**E**) rs713065(C) down-regulates FZD4 protein expression, as determined by Western blot with FLAG-labelled protein. Full length Western-blot images with different intensities were shown in supplementary Fig. S1.

To evaluate whether the change from T to C at rs713065 affects *FZD4* expression, partial FZD4 3′UTRs containing the variant (C) and wildtype (T) alleles for rs713065 were cloned into GFP reporter plasmids and transfected into H1299 cells, as shown in Fig. 1A. There was a significant decrease in GFP expression in H1299 cells transfected with SNP-3′UTR compared with those transfected with WT-3′UTR after 1, 2, and 3 hours (h). Furthermore, we detected that GFP expression was significantly decreased in cells from H1299, H226, A549, H661, and H1975 lung cancer cell lines transfected with GFP-SNP-3′UTR compared with cells that were transfected with GFP-WT-3′UTR (Fig. 1B). As an additional test for rs713065 SNP regulation, dual-luciferase vectors containing either the C or T allele at rs713065 were transiently transfected into H1299 cells, and relative firefly and sea pansy luciferase activity was measured. Relative luciferase activity was reduced 38% for the C allele relative to the T allele at rs713065 (Fig. 1C).

To further determine whether the rs713065 SNP may regulate *FZD4* expression, we generated FZD4 expression plasmids with 3′UTRs containing either the C or T allele of rs713065 and transfected them into H1299 cells. Expression of FZD4 carrying rs713065(C) was significantly down-regulated relative to rs713065 (T) 48 h after transfection, as measured by qRT-PCR (Fig. 1D). Down-regulated FZD4 protein expression in FZD4-SNP transfectants was also confirmed by Western blot (Fig. 1E). These findings support the hypothesis that the rs713065 variant allele may alter host gene expression and biological function as demonstrated by the down-regulated *FZD4* expression by the rs713065 C variant allele compared to the T wild-type allele.

### rs713065(C) Mediates FZD4 mRNA Transcript Cleavage

**Figure 2:**
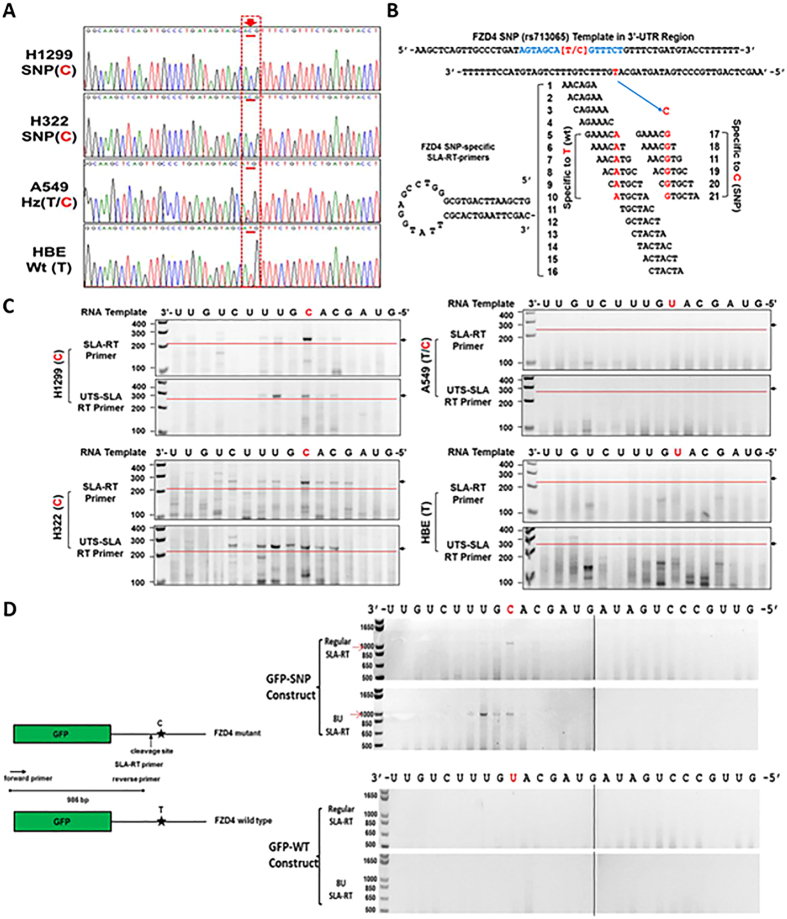
Cleavage and 3′-uridylation of endogenous FZD4 mRNA at miR-SNP loci detected by SLA-RT-PCR. **(A)** Identification of rs713065 alleles in normal Human Bronchial Epithelial Cell (HBEC) and NSCLC cells by automated DNA sequencing. H1299 and H322 cells carry two copies of the variant C allele (C/C), A549 cells carry one copy of the variant allele (T/C), and HBEC cells are homozygous for the wildtype allele (T/T). **(B)** FZD4-SNP specific SLA-RT primers. SLA-RT primers were designed to specifically detect the cleavage sites in the 3′UTR of FZD4 mRNA surrounding the SNP locus. 8U-tract-specific (8UTS)-SLA-RT primers corresponding to each SLA-RT primer were also made to detect 3′-uridylation of the cleaved mRNA fragments. **(C)** Validation of FZD4 mRNA cleavage at specific miRNA binding sites. SLA-RT-PCR detected FZD4 mRNA cleavage and 3′-uridylation in H1299 and H322 cells carrying the C allele, but not in A549 and normal HBEC cells carrying the T allele. The specific SLA-RT-PCR products (ranging in sizes from 236 bp to 220 bp, 3′ to 5′ on the FZD4 mRNA) were separated on a 2% agarose gel (for full length gel images see supplementary Fig. S2). **(D)** Differential mRNA cleavage in GFP-SNP-3′UTR and GFP-WT-3′UTR transfected cells. SLA-RT-PCR detected cleaved mRNA fragments of the predicted sizes in GFP-SNP-3′UTR transfected cells, compared with no cleaved mRNA in those transfected with GFP-WT-3′UTR. Gel images were cropped from full length individual gel images shown in supplementary Fig. S3.

By using a novel and sensitive SLA-RT-PCR assay developed in our laboratory^31^, we detected *FZD4* mRNA cleavage activity based on its 3′ terminal sequences in the FZD4-SNP bearing NSCLC cells. A series of SLA-RT primers with a 6-nucleotide (nt) probe at their 5′ termini was designed to match along the entire rs713065 sequences as well as sequences in their 5′- and 3′-adjacent regions for the initial RT reaction (Fig. 2B). By genotyping rs713065 status in lung cancer cell lines, we showed that H1299 and H322 cells have a C allele, A549 cells have a C/T allele, and human bronchial epithelial (HBE) cells have a T allele (Fig. 2C). SLA-RT-PCR detected *FZD4* cleavage in H1299 and H322 cells, which have an endogenous C allele at rs713065. There are no *FZD4* mRNA cleavage fragments at rs713065 in A549 cells (C/T) or in HBE cells, which have a wild-type T allele at rs713065 (Fig. 2c), displaying a differential regulation of endogenous FZD4 gene expression by the SNP variant.

It has been shown that non-templated oligouridines with various lengths (U-tracks) could be posttranscriptionally added to the 3′-ends of miRNA cleaved 5′ mRNA fragments after miRNA cleavage^32^. To determine whether oligouridines were added to the cleaved 5′ fragments of cleaved FZD4 transcripts accumulated around the rs713065 site, we modified SLA-RT primers by adding a track of varied numbers of adenosines at the 5′ end of the probe sequences in SLA-RT primers to match the non-templated oligouridine that could be potentially added to the 3′ ends of cleaved mRNA fragments. Significantly elevated amplicon intensities were detected in oligouridylated mRNA fragments in H1229 and H322 cells, suggesting the uridylation activity in the 3′UTR region containing the rs713065 C allele (Fig. 2C). To confirm this finding, we also used SLA-RT-PCR to detect GFP mRNA transcript cleavage and uridylation activity after H1299 cells were transfected with GFP-WT-3′UTR and GFP-SNP-3′UTR reporters. After 48 h of transfection, the cleaved and uridylated fragments of GFP mRNA were detected in cells transfected with GFP-SNP-3′UTR but not in those transfected with GFP-WT-3′UTR SLA-RT-PCR (Fig. 2D). These observations imply the potential of miRNAs that could specifically interact with the SNP-bearing gene transcripts, mediate mRNA cleavage, uridylation, degradation, and thus selectively silence expression of host genes and regulate their biological activities.

### miR-204 Differentially Regulates FZD4 Expression in rs713065 SNP Variants

**Figure 3:**
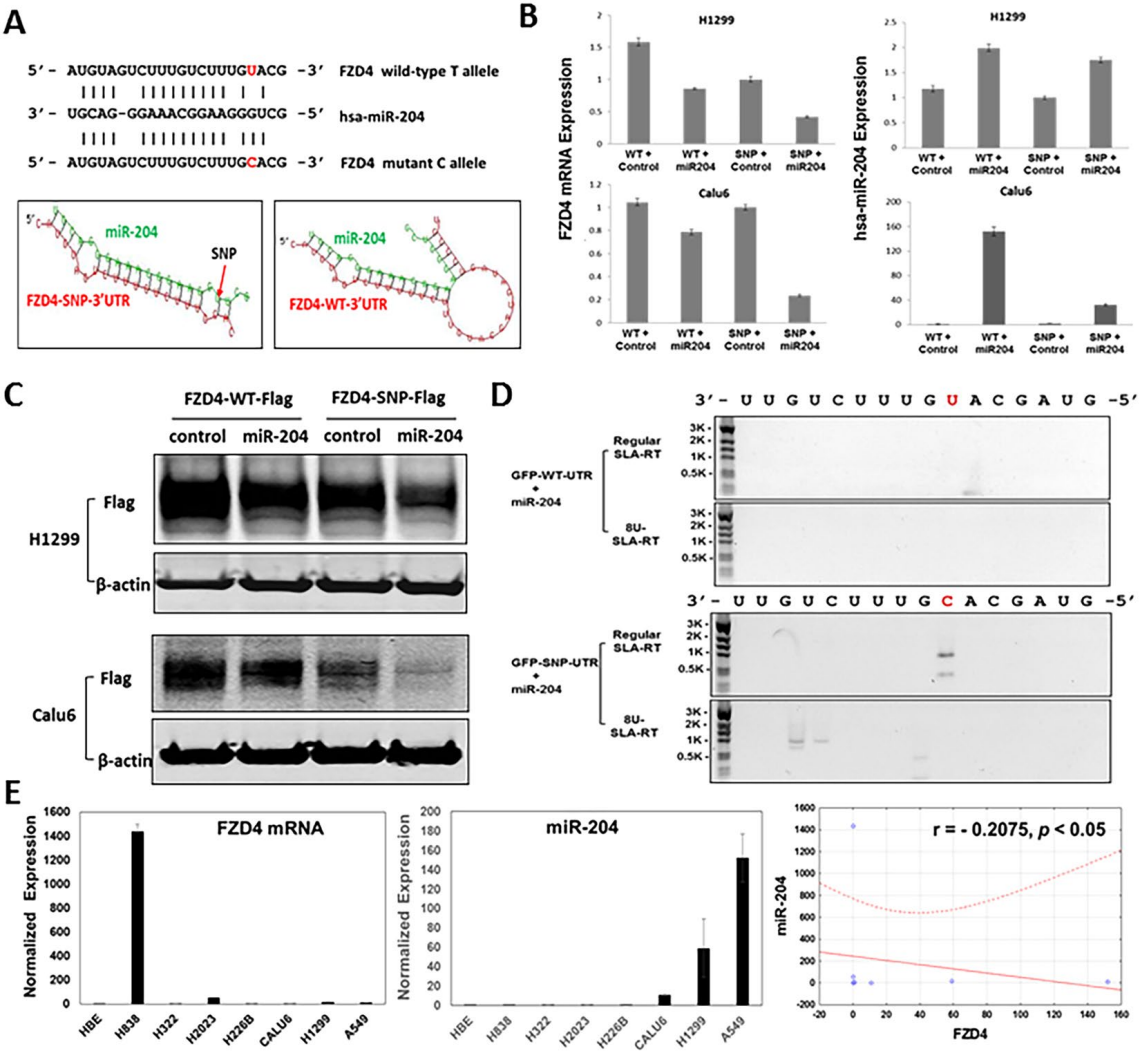
The differential regulation of target binding and gene expression in FZD4 3′UTR at the allelic variants of rs713065 loci by miR-204. **(A)** The complementary sequences and predicted secondary structures of miR-204 to FZD4-SNP and WT allelic variants at rs713065. At rs713065, the SNP variant C Watson-Crick base-pairs with G (shown in red) and favorable miRNA:mRNA binding pattern and secondary strucure, while the wildtype U allele wobble base-pairs with G. **(B)** Over-expression of miR-204 reduces intracellular levels of the FZD4-SNP-3′UTR mRNA, as determined by quantitative RT-PCR. **(C)** Over-expression of miR-204 leads to down-regulation of FZD4 protein in FZD4-Flag-SNP transfected H1299 cells, as determined by Western blot (see supplementary Fig. S4 for full length gel images). **(D)** miR-204 selectively induces SNP-3′UTR mRNA transcript cleavage, as detected by specific SLA-RT-PCR. (**E**) Expression of endogenous miR-204 and FZD4 and their correlation in NSCLC cell lines by a quantitative real-time RT-PCR analysis. A significant correlation (r = −0.2075, p < 0.05) between the endogenous mature miR-204 and the FZD4 mRNA expression was demonstrated by a Pearson’s correlation analysis.

The transcripts originated from SNP rs713065 luci in the 3′UTR of FZD4 display potential complementary miRNA biding sites. We performed various computational modeling of miRNA interactions with the linear and secondary structures of FZD4-SNP variant-containing transcripts in the 3′UTR region compared to those of the wild-type allele-containing transcripts. We identified the miR-204 as a potential candidate that could directly interact with the 3′UTR region in the FZD-SNP transcript. We predicted that miR-204 should target the site containing rs713065(C) by a higher affinity with over 14 consecutive complementary nucleotides, a lower relative free energy (rfe = −22.3 kcal/mol) in the microenvironment, and a more favorable miRNA:mRNA interaction secondary structure than the one containing rs713065 Wild-type (T) allele (Fig. 3A). We hypothesized that miR-204 would selectively target and cleave transcripts containing the C allele in the FZD4 3′UTR. To test this hypothesis, we made a pre-miR-204 expression plasmid and transiently transfected it into H1299 cells. A significant increase in mature miR-204 expression was detected in H1299 and Calu-6 cells transfected with pre-miR-204, as shown by qRT-PCR (Fig. 3B). The miR-204 and FZD4-WT-3′UTR or the miR-204 and FZD4-SNP-3′UTR vectors were co-transfected in H1299 and Calu- cells; miR-204 resulted in a 60% reduction in FZD4-SNP-3′UTR mRNA expression in H1299 cells and an 80% reduction in FZD4-SNP-3′UTR mRNA expression in Calu-6 cells, as assessed by qRT-PCR. In contrast, miR-204 resulted in a 40% reduction in FZD4-WT-3′UTR mRNA expression in H1299 cells and a 20% reduction in FZD4-WT-3′UTR mRNA expression in Calu-6 cells (Fig. 3B). Western-blotting confirmed that miR-204 inhibited FZD4 protein expression more strongly for FZD4-SNP-3′UTR than for FZD4-WT-3′UTR in both H1299 and Calu6 cells (Fig. 3C). The miR-204 also induced GFP mRNA transcript cleavage after co-transfection with GFP-SNP-3′UTR, suggesting that miR-204 specifically induced rs713065(C)-bearing transcript cleavage and degradation, as confirmed by SLA-RT-PCR (Fig. 3D). Furthermore, the endogenous expression of mature miR-204 was reciprocally correlated with the level of FZD4 mRNA transcription (r = −0.2075, *p* < 0.05) in a panel of selected NSCLC cell lines as demonstrated by a real-time qRT-PCR analysis (Fig. 3E). Similarly, a down-regulated expression of miR-204 was also detected in a panel of ~40 NSCLC cell lines compared to that of normal human bronchial epithelial (HBEC) cells (see Supplementary Fig. S5A) and a reciprocal correlation between miR-204 and FZD4 gene expression was displayed in primary lung adenocarcinoma (LUAD) and squamous carcinoma (LUSC) tumors from The Cancer Genome Atlas (TCGA) database (see Supplementary Fig. S5B). These results imply the potential role of miR-204 as a tumor suppressor miRNA and in regulation of FZD4 gene expression in NSCLC.

### rs713065(C) Inhibits Lung Cancer Cell Colony Formation and Migration

**Figure 4:**
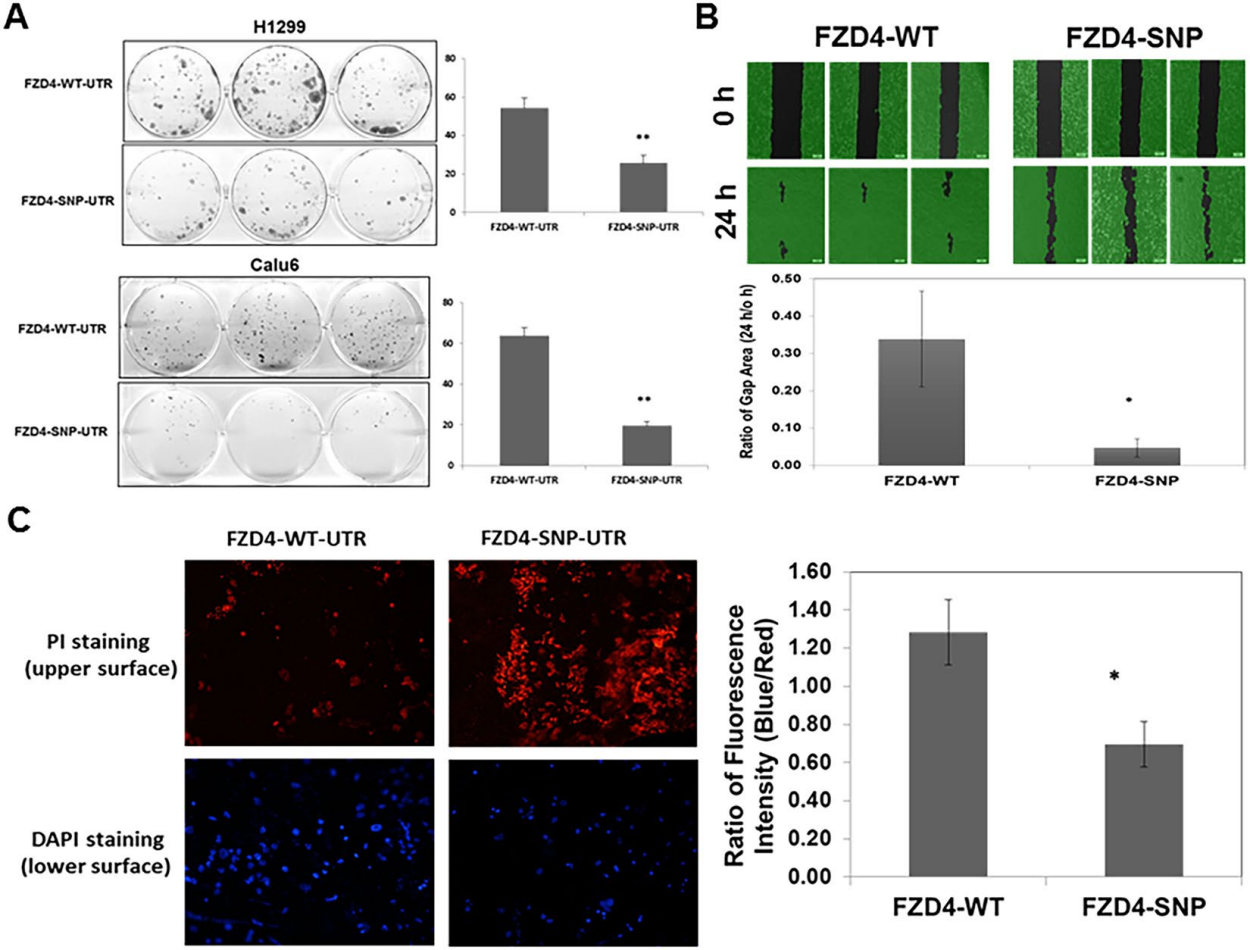
Inhibition of tumor colony formation and cell migration by FZD4-miR-SNP in NSCLC cells. **(A)** The rs713065(C) allele reduces tumor cell induced-clonogenesis in C/C homozygous H1299 cells as well as C/T heterozygous Calu6 cells transfected with the FZD4-3′UTR-SNP plasmid, compared with those transfected with the FZD4-3′UTR-WT expression plasmid. *p < 0.05; **p < 0.001. **(B)** FZD4-3′UTR-SNP inhibits H1299 cell mobility compared with mobility in cells transfected with the FZD4-3′UTR-WT plasmid. Wound healing of cultured H1299 cells using phase contrast. Photomicrographs were taken at 0 h and 36 h. **(C)** Inhibition of tumor cell migration/invasion in H1299 cells transfected with FZD4-3′UTR-SNP or FZD4-3′UTR-WT plasmids by a FluoroBlock membrane assay in transwell Boyden chambers.

To further characterize the biological significance of rs713065(C), we assessed the effect of the C allele on colony forming ability in NSCLC. We found that FZD4-SNP-3′UTR over-expression in H1299 and Calu-6 cells significantly inhibited these tumor cell-induced colony formation compared with FZD4-WT-3′UTR over-expression (Fig. 4A). We further assessed the effect of the C allele on lung cancer cell migration using the wound-healing assay. Cell migration was inhibited in H1975 cells after FZD4-SNP-3′UTR over-expression relative to FZD4-WT-3′UTR over-expression (Fig. 4B). To quantitatively evaluate the effects of rs713065 on invasion, we used a fluorescence-based *in vitro* invasion assay. FZD4-SNP-3′UTR over-expression in H1299 cells showed a 50% reduction in tumor cell invasion compared with FZD-WT-3′UTR over-expression (Fig. 4C).

### rs713065(C) Modulates Wnt Signaling through Down-Regulation of FZD4

**Figure 5:**
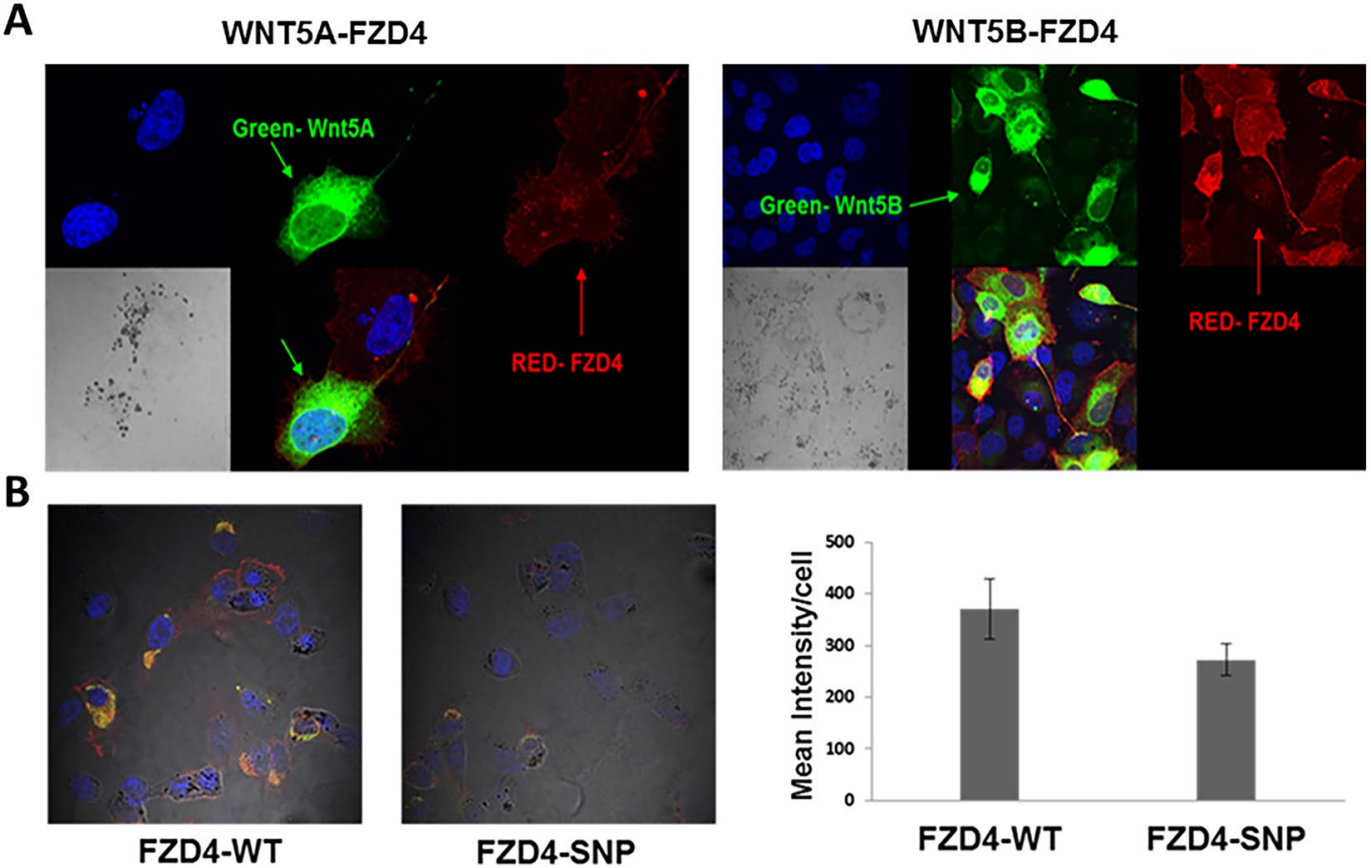
Modulation of FZD4 expression by WNT and FZD4 protein interaction in rs713065 variants in H1299 cells. **(A)** Interaction of Wnt ligands and FZD4 receptor in H1299 cells transfected with Wnt-5A-V5 and FZD4-FLAG expression plasmids shown by confocal fluorescence imaging. **(B)** rs713065 (C) down-regulates FZD4, as quantitatively measured by confocal microscopy.

To determine the role of rs713065-mediated FZD4 expression in Wnt signaling, we first examined the interaction between Wnt ligands and the FZD4 receptor. H1299 cells were co-transfected with Wnt-V5 and FZD4-FLAG tagged plasmids and analyzed by confocal fluorescence microscopy. The results showed co-localization of Wnt-5A/FZD4 and Wnt-5B/FZD4, suggesting interaction between these Wnt ligands and the FZD4 receptor (Fig. 5A). In addition, down-regulation of FZD4 expression by the C allele of rs713065 relative to the T allele was confirmed by a quantitative con-focal immunofluorescence imaging analysis (Fig. 5B).

**Figure 6:**
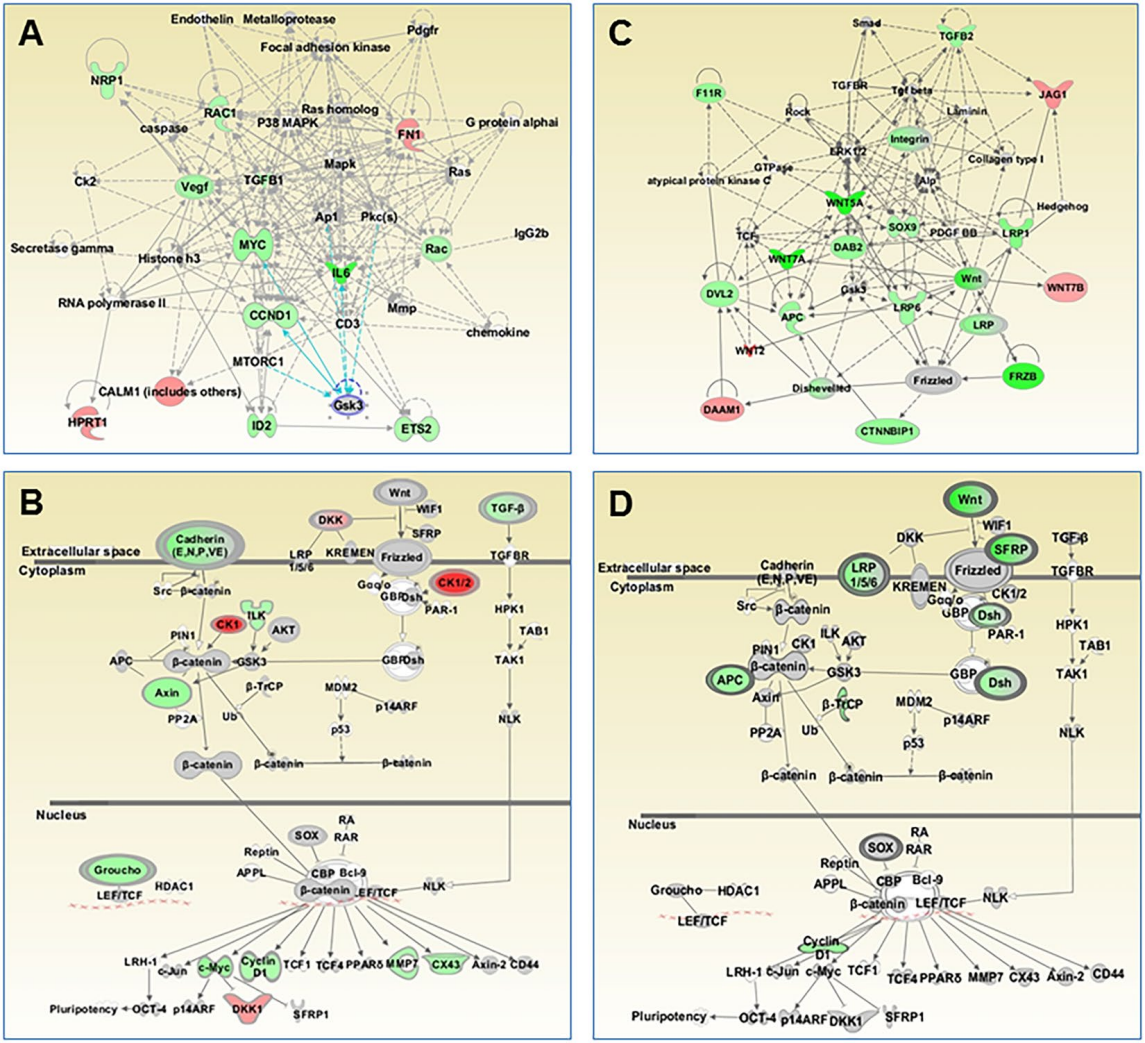
Modulation of Wnt/FZD4 signaling pathways through the interaction of miR-204 with FZD4-miR-SNP (rs713065) variant in NSCLC cells. **(A)** The top-scored network in Cell Cycle, Gene Expression, and Cellular Function and (**B**) canonical Wnt/β-Catenin signaling pathway associated with FZD4-miR-SNP (rs713065) and Wnt5A activities in H1299 cells were representatively displayed. H1299 cells were transfected with either FZD4-SNP-3′UTR or FZD4-WT-3′UTR. After incubation for 48 h, the total RNAs were isolated and absolute gene expression profiles were determined by nCounter, with differentially expressed pathways identified through Ingenuity® Pathway Analysis. **(C)** Modulation of top-scored cellular network and (**D**) Wnt/β-Catenin canonical pathways through the miR-204 targeted FZD4 expression in 3′UTR containing FZD4-miR-SNP. Key molecules identified through NanoString and IPA analysis (red if is up-regulated in H1299 cells co-transfected with FZD4-SNP/Wnt-5a compared to FZD4-WT/Wnt-5a in the presence or absence of the ectopic expression of miR-204, green if is down-regulated). The intensity of the color indicates expression level or degree of regulation. The top-scored networks and signaling pathways and their associated molecules and cellular functions were summarized in details in Supplementary Tables 1–5)

To determine if there is any alteration of Wnt networks and signaling pathways in the presence of FZD4-SNP rs713065 in NSCLC cells, we performed a quantitative analysis of Wnt/EMT-pathway-associated gene expression using a custom-designed Wnt/EMT gene code set containing ~200 Wnt and EMT signaling pathway-associated genes and internal house-keeping gene controls (Supplementary Table 1) by a NanoString nCounter, results were statistically analyzed for significant changes among treatment groups, and the significantly-altered expression of genes were applied to characterization of biological pathways and networks using Ingenuity Pathway Analysis (IPA^®^, QIAGEN, Redwood City) (Fig. 6). NSCLC A549 cells were co-transfected with Wnt-5A and FZD4-WT-3′UTR or Wnt-5A and FZD4-SNP-3′UTR and the total RNAs were prepared from these cell transfectants for the absolute quantification of gene expression. The results showed that the rs713065/C allele significantly altered Wnt/Catenin signaling (*p*-Value = 2.19E-16). The results of IPA were summarized in Supplementary Table 2. The five top WNT pathway regulators identified by IPA and downregulated by the rs713065/C allele were: *NOTCH1* (p-Value = 4.00E-26), *ERBB2* (p-Value = 1.57E-25), *STAT3* (p-Value = 7.60E-25), *CTNNB1*(p-Value = 8.27E-25), and *HRAS* (p-Value = 6.46E-24) (Table 1 and Supplementary Table 1). These gene products are all regulators of the Wnt signaling pathway or pathways regulated by the Wnt signaling. The top-1 network (Fig. 6A) and canonical Wnt/β-Catenin signaling pathways (Fig. 6B) associated with FZD4-miR-SNP and Wnt5A activities in H1299 cells by IPA was representatively displayed (see Supplementary Tables 2, 4, and 5 for their associated molecules and biological functions).

**Table 1:** The five top WNT pathway regulators identified by IPA and downregulated by the rs713065/C allele (see Supplementary Table 2 for IPA summary).

Upstream Regulator	*p*-Value of overlap	Predicted Activation
NOTCH1	4.00E-26	Inhibited
ERBB2	1.57E-25	Inhibited
STAT3	7.60E-25	Inhibited
CTNNB1	8.27E-25	Inhibited
HRAS	8.48E-24	Inhibited

Next, we determined if miR-204 targeting at rs713065 SNP can regulate the Wnt/Catenin signaling pathway. H1299 cells were transfected with miR-204 and FZD4-SNP-3′UTR and compared to non-specific miR-control and FZD4-SNP-3′UTR. Similarly, the top-1 network (Fig. 6C) and canonical Wnt/β-Catenin signaling pathways (Fig. 6D) by IPA of quantitative expression data obtained from NanoString Wnt/EMT gene code-set assay were representatively shown, suggesting that miR-204 could differentially targete at FZD4-SNP-3′UTR and down-regulate key mediators of Wnt/Catenin signaling, including *Wnt*, *SFRP*, *LRP1/5/6*, *Dsh* and *cyclin D1* (Fig. 6C and D, and Supplementary Tables 3, 4, and 5).

## Discussion

NSCLC is one of the most aggressive cancers and is characterized by a very poor prognosis. A clear understanding of the genetic factors responsible for the development and recurrence of NSCLC may lead to the identification of novel drug targets. Because of their critical role in regulating gene expression, both miRNAs and miRNA binding sites are highly conserved. While variation in miRNA sequence is constrained due to structural considerations, allelic variations in miRNA binding sites could have significant functional implications^33^. We previously showed that the C allele of FZD4:rs713065 was significantly associated with decreased risk of death in NSCLC patients^30^. We hypothesized that this SNP could be functionally important in the progression of lung cancer. To test this hypothesis and elucidate the mechanism by which rs713065(C) acts, we characterized the regulatory function of the rs713065 SNP. We found that the rs713065 C allele significantly inhibited GFP and luciferase reporter gene and protein expression compared with the rs713065 T allele. Furthermore, SLA-RT-PCR showed that the C allele down-regulated FZD4 expression by modulating FZD4 mRNA transcript cleavage, 3′-uridylation, and mRNA stability. We detected endogenous FZD4 mRNA cleavage and 3′-uridylation of the resulting fragments in H1299 and H322 cells bearing the C allele, but not in A549 and normal HBEC cells bearing the T allele.

The involvement of miRNAs in human cancers has been extensively studied. Our analysis suggested that rs713065 C > T could be a target sequence for miR-204. In previous studies, miR-204 has been reported to play an important role in tumorigenesis, including regulation of carcinogenesis in peripheral nerve sheath tumors^34^, and migration and invasion of gastric cancer^35^. However, very little is known about mechanisms by which miR-204 regulates oncogenesis in lung cancers. Our SLA-RT-PCR assay revealed that miR-204 induced cleavage of GFP-SNP-3′UTR, but not the GFP-WT-3′UTR transcript, confirming a predicted functional interaction between miR-204 and the FZD4 3′UTR containing the rs713065 C allele. Taken together, our study revealed that miR-204 acted as a potent lung tumor suppressor by targeting the FZD4:rs713065 C allele, therefore inhibiting FZD4 expression. Importantly, our analysis demonstrated that miR-204 targeting at the FZD: rs713065 C allele inhibited the Wnt pathway key regulators, including WNT, SFRP, DSH, APC, and CYCLIN D1.

*FZD4* is a receptor for Wnt proteins, which are coupled to the β-catenin canonical signaling pathway and non-canonical pathways. NanoString data showed that the FZD4:rs713065 C allele inhibited the Wnt/Catenin signaling pathway through inhibition of *FZD4* expression. WNTs and their downstream effectors regulate various processes that are important for cancer progression. WNTs modulate both CTNNB1-dependent (canonical) WNT signaling and CTNNB1-independent (non-canonical) WNT signaling pathways. In our studies, IPA identified the top five Wnt pathway regulators downregulated by the rs713065: C allele included *NOTCH1*, *ERBB2*, *STAT3*, *CTNNB1*, and *HRAS*. Wnt and Notch signaling pathways are two major channels of communication that control cell proliferation and migration, which are closely intertwined during tumorigenesis. An increasing number of studies have shown that *NOTCH1* plays a critical role in EMT^36^. Inhibition of *NOTCH1* may also suppress NSCLC cell invasion and metastasis. In addition, overexpression of *ERBB2* is strongly associated with increased recurrence and poorer prognosis for NSCLC. The rs713065/C allele down-regulated *ERBB2*, which suggests an association with a lower recurrence rate for NSCLC. Persistent phosphorylation of *STAT3* has been observed in 22–65% of NSCLC and is associated with a poor prognosis. Overexpression of *STAT3* was associated with chemoresistance and radioresistance in NSCLC cells^37^. Importantly, *CTNNB1* plays an essential role in Wnt/Catenin signaling. The *CTNNB1* encoded protein β-catenin accumulates in the nucleus and serves as a coactivator for TCF to activate Wnt-target gene transcription^38^. Numerous studies indicate that Wnt–CTNNB1 signaling contributes to cancer progression through the maintenance of highly tumorigenic tumor-initiating cells^39^. Loss of CTNNB1 also reduces cell tumor-forming abilities. In the past decade, there was an explosion in the development of strategies for targeting Wnt-CTNNB1 signaling, including small molecules, antibodies and peptides. However, inhibiting Wnt-CTNNB1 signaling pathway alone is unlikely to succeed in anti-cancer treatment owing to the co-activation of numerous oncogenic pathways in most cancers. Importantly, further studies aimed at identifying the genetic factors predicting responses to treatment with Wnt-CTNNB1 signaling will be important in determining potential new therapies. SNP rs713065(C) in the miR-204 binding site in the FZD4 3′UTR might shed light on targeting at Wnt-CTNNB1 signaling anti-cancer treatment.

In summary, our results provide first-handed connection of novel epidemiological miR-SNP biomarkers in 3′UTR region of host genes to their biological function, cellular process, molecular mechanism, and clinical relevance in NSCLC pathogenesis and prognosis. Our findings suggest that the novel epidemiological miR-SNP (rs713065) loci in the 3′UTR of FZD4 may significantly influence overall survival in early stage NSCLC patients by specifically and differentially interacting with miRNAs such as miR-204, modulating FZD4 expression and cellular function in the Wnt/FZD4-signaling driven tumor cell proliferation and progression pathways thus identifying novel therapeutic targets and treatment strategies for NSCLC.

## Methods

### Biological Function of FZD4-miR-SNP and associated miRNA

We used FZD4 or reporter gene expression system to determine how these Wnt-SNP- associated miRNAs differentially regulate their host/target gene expression, cellular function, and clinical relevance of this novel epidemiological FZD4-miR-SNP biomarker in NSCLC cell lines, preclinical mouse models, and in clinical plasma and tissue samples. Additional details are provided in *SI Materials and Methods*.

### SLA-RT-PCR Assay

We used a novel stem-loop array-reverse transcription-PCR (SLA-RT-PCR) assay to assess miRNA:target mRNA interaction at the specific FZD4-miR-SNP locus and detected miRNA-mediated FZD4 mRNA cleavage and 3′-uridylation in FZD4-SNP (rs713065) mutant (C) or wild-type (T) allele-bearing NSCLC cell lines. Total mRNA was isolated using TRIzol reagent (Invitrogen, Carlsbad, CA); additional phenol:chloroform extraction was performed before ethanol precipitation according to the manufacturer’s instructions. The principle and applications of SLA-RT-PCR method was previously described in details^32, 40^. and in *SI Materials and Methods*.

### NanoString Analysis

Tumor cells were harvested by centrifugation at designated time points and a custom-designed CodeSet was used to quantitatively measure expression of 209 Wnt and TMT signaling-related genes (see *S* Table 1 for the gene list). Testing samples were purified using an nCounter Prep Station and scanned on an nCounter Digital Analyzer; data were extracted using an nCounter RCC Collector (NanoString Technologies, Seattle, WA). The significant gene expression data sets were submitted for biological pathway and signaling network analysis using Ingenuity Pathway Analysis software (IPA^®^, QIAGEN Redwood City). NanoString assay and IPA data analysis were described in details in *SI Materials and Methods and* Supplemental Table 1–5).

### Statistical Analysis

Quantitative variables were reported as median ± SD. Differentially expressed genes between two groups were analyzed by two sided *t* tests. Benjamini-Hochberg method was used to adjust for multiple hypothesis testing, and generate false discovery rate (FDR q values). Top genes (corresponding FDR q values range from 0.029 to 0.493, p values range from 0.0001 to 0.0738) were selected for pathway analysis using Ingenuity Pathway Analysis software (http://www.ingenuity.com/). Data analyses were performed using R packages (https://www.r-project.org/), a publically available statistical computing tool.

## Electronic supplementary material

Supplementary Information
