# Supplementary material for: A miR-SNP biomarker linked to an increased lung cancer survival by miRNA-mediated down-regulation of FZD4 expression and Wnt signaling
Source: Sci Rep. 2017 Aug 22;7:9029. doi: 10.1038/s41598-017-09604-4 (PMC5567228; doi:10.1038/s41598-017-09604-4)

Supplementary Information

A miR-SNP biomarker linked to an increased lung cancer survival by miRNA-mediated down-regulation of FZD4 expression and Wnt signaling

Jing Lin, Roza Zandi, Ruping Shao, Jian Gu, Yuanqin Ye, Jing Wang, Yang Zhao, Alexander Pertsemlidis, Ignacio I. Wistuba, Xifeng Wu, Jack A. Roth, and Lin Ji

Supplementary Information

Supplementary Materials and Methods

Cell Culture and Plasmid Transfection. Human NSCLC cell lines H1299, A549, H322, Calu6, H661 and H1975 were obtained from the American Type Culture Collection (Manassas, VA) and cell identities were verified. Cells were grown in RPMI 1640 supplemented with 10% fetal bovine serum (FBS) in an atmosphere of humidified air containing 5% CO_2. Immortalized normal human bronchial epithelial (HBE) cells were obtained from Dr. John Minna's laboratory at The University of Texas Southwestern Medical Center, Dallas. Human bronchial epithelial cells (HBECs) were grown in Keratinocyte-SFM medium (Thermo Fisher Scientific) in an atmosphere of humidified air containing 5% CO_2. For transfections, plasmid DNA was mixed with an equal volume of 8 mM DOTAP:Cholesterol (DC) liposome nanoparticles to make DC:DNA complexes. DC:DNA nanoparticles was prepared as described previously (41). Cells were transfected with 2 μL DC nanoparticles and 2 μg plasmid DNA, and incubated for the designated periods of time.

Plasmid Construction. We generated FZD4-WT-3′UTR and FZD4-SNP-3′UTR plasmids as follows: The FZD4 coding region and 343-bp 3′UTR were amplified from human genomic DNA and then subcloned into a clinically approved plasmid vector with an expression cassette consisting of a CMV promoter and BGH polyA sequences at KpnI/PmeI restriction sites. Reporter plasmids GFP-WT-3′UTR and GFP-SNP-3′UTR were constructed by inserting WT-3'UTR and SNP-3′UTR sequences of the GFP gene reporter gene. In addition, the 343-bp variant and wildtype FZD4 3′UTR regions were also cloned downstream of the firefly luciferase gene in pmirGLO Dual-Luciferase miRNA Target Expression Vector (Promega, Madison, WI). All plasmid constructs were confirmed by sequencing.

Cell Proliferation Assay. The effect of FZD4-WT-3′UTR or FZD4-SNP-3′UTR on cell proliferation was determined by using the XTT assay (Roche Diagnostics, Indianapolis, IN). Cells were plated in 96-well plates at 1×10^4 cells/well. After 24 h of incubation under standard conditions, cells were transfected with FZD4-WT-3′UTR or FZD4-SNP-3′UTR plasmid. Cell viability was assessed by a microplate reader (BioTek ELx808, Winooski, VT).

Wound Healing Assay. H1975 cells were transfected with either FZD4-WT-3′UTR or FZD4-SNP-3′UTR plasmid. After reaching confluence, a 1 mm strip was removed with a standard 200-μL pipette tip (42). The wounded monolayers were washed by PBS to remove non-adherent cells. Pictures were taken immediately after non-adherent cell removal and after 48 and 96 h.

Cell Colony Formation Assay. H1299 and Calu6 cells were plated at a density of 200 cells/well in 6-well plates. After 24h, H1299 cells and Calu6 cells were transfected with FZD4-WT-3′UTR plasmid or FZD4-SNP-3′UTR plasmid and cultured in medium containing 500 µg/mL G418, refreshing every 3 days. After two weeks of treatment, cell colonies were stained with crystal violet (0.1% in 20% methanol).

Cell Migration Assay. The migration ability of H1299 cells transfected with FZD4-WT-3′UTR or FZD4-SNP-3′UTR plasmid was evaluated in a transwell Boyden chamber with a 8 µm FluoroBlock™ membrane (BD Biosciences) to block the passage of light and allow for monitoring and quantification of migrating cells. After 24 h of transfection, cells in FBS-free RPMI 1640 were added to the upper chamber ($4\text{x}10^4$ cells/well). At the same time, 0.5 mL of RPMI 1640 with 10%

FBS was added to the lower chamber. After incubation for 24 h, cells remaining on the upper surface of the filter membrane were stained with PI, and cells on the lower surfaces were stained with DAPI. Images were captured using a DXM1200 microscope (Nikon, Japan).

Dual-Luciferase Assay. H1299 cells were transfected with Dual-Luc-SNP-3′UTR or Dual-Luc-WT-3′UTR reporter plasmids. 48 h after transfection, cells were lysed to measure firefly and Renilla luciferase activities by the Dual-Luciferase Reporter Assay System (Promega). Relative luciferase activity was calculated by normalizing firefly luciferase activity to Renilla luciferase activity. Each experiment was repeated three times.

SLA-RT-PCR Assay. Total mRNA was isolated using TRIzol reagent (Invitrogen, Carlsbad, CA); additional phenol:chloroform extraction was performed before ethanol precipitation according to the manufacturer's instructions. The mRNAs were treated briefly with 0.04 U/µL RNase-free DNase I (New England Biolabs, Ipswich, MA). The mRNA was reverse-transcribed by using a High Capacity Reverse Transcription Kit (Life Technologies, Carlsbad, CA) in combination with ST-RT-primer. 20 µL of the RT reaction contained 50 ng of total RNA, $5x10^{-12}$ mol of SLA-RT primer, 2 µL of 10X RT buffer, 1 µL of MultiScribe Reverse Transcriptase, and 0.8 µL of 100 mM dNTPs. The nucleotide sequences of the SLA-RT primers are listed in Fig. 2B. To increase reverse transcription efficiency, a pulsed RT reaction was performed on a DNAEngine Peltier Thermal Cycler (Bio-Rad) with 60 cycles of 18°C for 1 min and 37°C for 1 s, followed by 60 cycles of 20°C for 1 min, 37°C for 1 s, 37°C for 30 min, 42°C for 20 min and 85°C for 10 min, and then holding at 4°C. RT products were then subjected to further PCR evaluation. The PCR primer sequence was as follows: Sense Primer: 5′-TCCTACAAGGATCAGATACTGGAG-3′. Antisense

primer: 5′-GTGCGGGTCCGAGGTATTC-3′. The PCR was run at 95°C for 5 min, followed by 35 cycles of 95°C for 30 s, 60°C for 30 s, and 72°C for 30 s. PCR products were analyzed by agarose gel electrophoresis in 1X Tris-borate-EDTA (TBE) buffer. Electrophoresis was performed at 100 volts for 60 min. The gel was stained in an ethidium bromide bath for 10 min before visualization with a UV transilluminator (AlphaImager HP, Protein Simple, San Jose, CA). The principle and applications of SLA-RT-PCR method was described in detailed.(32, 40)

Real-Time PCR Assay. Total mRNA was reverse-transcribed into cDNA by using an oligo (dT) primer and High Capacity Reverse Transcription Kit (Life Technologies). To quantify FZD4 mRNA, PCR was performed in triplicate in 10 µL reaction volumes with TaqMan Universal PCR Mix (Applied Biosystem, Foster City, CA). Reactions were incubated at 95°C for 10 min, followed by 40 cycles of 95°C for 30 s, 60°C for 30 s, and 72°C for 30 s. Ct values were determined by setting a fixed threshold. The relative amount of FZD4 mRNA was normalized to GAPDH using the $2^{-\Delta\Delta Ct}$ method as instructed by the manufacturer.

Western Blot Assay. Cells were collected 48 h after transfection and lysed with Laemmli urea buffer. Cell lysates were separated by 10% SDS PAGE gel and transferred to nitrocellulose membranes for conventional western blot analysis. Membranes were probed with following antibodies: anti-Flag (Sigma Aldrich, #F3165), anti-β-actin (Sigma Aldrich, #A2228), IRDye® 800CW goat anti-rabbit (LICOR, #926-32211), and IRDye® 680RD goat anti-mouse (LICOR, #926-68070). Protein levels were normalized with β-actin.

NanoString Analysis. Tumor cells were harvested by centrifugation at designated time points and

10,000 cells were re-suspended in 1.0 μL of Qiagen RLT lysis buffer and hybridized at 65°C for 16 h to a custom-designed CodeSet to quantitatively measure expression of 209 Wnt and EMT signaling-related genes (see **Supplemental Table 1** for the gene list). Testing samples were purified using an nCounter Prep Station and scanned on an nCounter Digital Analyzer; data were extracted using an nCounter RCC Collector (NanoString Technologies, Seattle, WA). Analysis of raw mRNA counts was performed using nSolver software. Data were normalized against 6 positive control oligonucleotides, 8 negative control oligonucleotides, and 5 housekeeping genes. All target gene expression were analyzed using the DE (differential expression) call model (NanoString), derived from a large experiment in which multiple technical replicates were run on numerous samples and CodeSets, creating a rule mapping any given raw expression value to the 95% of technical variability of genes at that expression level.

Immunostaining Assay. H1299 cells were co-transfected with Flag-FZD4 and either Wnt5A-V5 or Wnt5B-V5 plasmid (Open Source Wnt Project Plasmids) (43). After 24 h of transfection, cell were fixed with 4% paraformaldehyde in PBS and permeabilized with methanol for 5 min. Cells were incubated with anti-Flag (1:50 dilution, Sigma Aldrich, #F3165) and anti-V5 (1:50 dilution, Sigma Aldrich, #V8137) for 1 h at RT. After washing with PBS, cells were incubated with Alexa Fluor 549-conjugated secondary antibody (1:650 dilution) and Alexa Fluor 488-conjugated secondary antibody (1:650 dilution) for 1 h at RT. After additional washing, cells were counterstained with DAPI. Images were captured on an Olympus IX81 DSU confocal microscope. Images are representative of two independent experiments, each performed three times.

Statistical Analysis. Quantitative variables were reported as median ± SD. Differentially

expressed genes between two groups were analyzed by two sided *t* tests. Benjamini-Hochberg method was used to adjust for multiple hypothesis testing, and generate false discovery rate (FDR q values). Top genes (corresponding FDR q values range from 0.029 to 0.493, p values range from 0.0001 to 0.0738) were selected for pathway analysis using Ingenuity Pathway Analysis (IPA) software (http://www.ingenuity.com/). Summaries of IPA are presented in **Supplemental Table 2** and **3**. Data analyses were performed using R packages (https://www.r-project.org/), a publically available statistical computing tool.

Supplementary Table 1. WNT-EMT Signaling Pathway Gene Code Set for NanoString Analysis.

Gene Name	Accession #	Class Name
ABCB1	NM_000927.3	Endogenous
AHNAK	NM_001101.2	Endogenous
AHR	NM_001620.2	Endogenous
AKT1	NM_001621.3	Endogenous
ANGPTL4	NM_001014432.1	Endogenous
ANTXR1	NR_104213.1	Endogenous
APC	NM_018153.3	Endogenous
AXIN1	NM_000038.3	Endogenous
AXIN2	NM_181050.1	Endogenous
BGLAP	NM_004655.3	Endogenous
BIRC5	NM_004048.2	Endogenous
BMP1	NM_199173.3	Endogenous
BMP2	NM_001168.2	Endogenous
BMP4	NM_001199.1	Endogenous
BMP7	NM_001200.2	Endogenous
BOD1	NM_001202.2	Endogenous
BTRC	NM_001719.1	Endogenous
CACNA2D3	NM_138369.1	Endogenous
CALD1	NM_001256856.1	Endogenous
CALM1	NM_018398.2	Endogenous
CAMK2N1	NM_004342.6	Endogenous
CAV2	NM_006888.3	Endogenous
CCND1	NM_018584.5	Endogenous
CCND2	NM_198212.1	Endogenous
CD44	NM_053056.2	Endogenous
CDH1	NM_001759.3	Endogenous
CDH2	NM_001001392.1	Endogenous
CDKN2A	NM_004360.2	Endogenous
CDON	NM_001792.3	Endogenous
CEBPD	NM_058195.2	Endogenous
CHSY1	NM_016952.4	Endogenous
COL1A2	NM_005195.3	Endogenous
COL3A1	NM_014918.4	Endogenous

**COL5A2**	NM_000089.3	Endogenous
**CSNK1A1**	NM_000090.3	Endogenous
**CTBP1**	NM_000393.3	Endogenous
**CTGF**	NM_001892.4	Endogenous
**CTNNB1**	NM_001328.2	Endogenous
**CTNNBIP1**	NM_001901.2	Endogenous
**CUBN**	NM_001098210.1	Endogenous
**CXADR**	NM_020248.2	Endogenous
**CYP4V2**	NM_001081.3	Endogenous
**DAAM1**	NM_001207063.1	Endogenous
**DAB2**	NM_207352.3	Endogenous
**DESI1**	NM_014992.1	Endogenous
**DKK1**	NM_001343.2	Endogenous
**DKK3**	XM_005261571.3	Endogenous
**DLK1**	NM_012242.2	Endogenous
**DPP10**	NM_001018057.1	Endogenous
**DSC2**	NM_003836.4	Endogenous
**DSP**	NM_001004360.3	Endogenous
**DVL1**	NM_024422.3	Endogenous
**DVL2**	NM_001008844.1	Endogenous
**EFNB1**	NM_004421.2	Endogenous
**EGFR**	NM_004422.2	Endogenous
**EP300**	NM_004429.4	Endogenous
**ERBB3**	NM_201282.1	Endogenous
**ESR1**	NM_001429.2	Endogenous
**ETS2**	NM_001005915.1	Endogenous
**F11R**	NM_000125.2	Endogenous
**FBXW11**	NM_005239.4	Endogenous
**FGF20**	NM_144503.1	Endogenous
**FGF4**	NM_033645.2	Endogenous
**FGF7**	NM_019851.1	Endogenous
**FGFBP1**	XM_005273847.1	Endogenous
**FN1**	NM_002009.3	Endogenous
**FOSL1**	NM_005130.3	Endogenous
**FOXC2**	NM_212482.1	Endogenous
**FRZB**	NM_005438.3	Endogenous
**FZD1**	NM_005251.2	Endogenous
**FZD2**	NM_001463.2	Endogenous
**FZD3**	NM_003505.1	Endogenous
**FZD4**	NM_001466.2	Endogenous
**FZD5**	NM_017412.3	Endogenous
**FZD6**	NM_012193.2	Endogenous
**FZD7**	NM_003468.2	Endogenous
**FZD8**	NM_003506.2	Endogenous
**FZD9**	NM_003507.1	Endogenous
**GDF5**	NM_031866.1	Endogenous
**GJA1**	NM_003508.2	Endogenous
**GNG11**	NM_002046.3	Endogenous
**GSC**	NM_000557.2	Endogenous
**GSK3B**	NM_000165.3	Endogenous
**HSPA12A**	NM_004126.3	Endogenous
**ID2**	NM_173849.2	Endogenous
**IGF1**	NM_002093.2	Endogenous
**IGF2**	NM_000194.1	Endogenous
**IGFBP4**	XM_048898.9	Endogenous
**IL6**	NM_002166.4	Endogenous
**ILK**	NM_000618.3	Endogenous
**IRS1**	NM_001127598.1	Endogenous
**ITGA5**	NM_001552.2	Endogenous
**ITGAV**	NM_000600.3	Endogenous
**ITGB1**	NM_004517.2	Endogenous

**JAG1**	NM_005544.2	Endogenous
**JUN**	NM_002205.2	Endogenous
**KLF5**	NM_002210.2	Endogenous
**KREMEN1**	NM_033666.2	Endogenous
**KRT14**	NM_000214.2	Endogenous
**KRT19**	NM_002228.3	Endogenous
**KRT7**	NM_001730.3	Endogenous
**LEF1**	NM_001039570.1	Endogenous
**LRP1**	NM_000526.4	Endogenous
**LRP5**	NM_002276.4	Endogenous
**LRP6**	NM_005556.3	Endogenous
**MAP1B**	NM_016269.3	Endogenous
**MAPK8**	NM_002332.2	Endogenous
**MET**	NM_002335.1	Endogenous
**MMP2**	NM_002336.1	Endogenous
**MMP3**	NM_005909.3	Endogenous
**MMP7**	NM_002750.2	Endogenous
**MMP9**	NM_001127500.1	Endogenous
**MSN**	NM_004530.2	Endogenous
**MST1R**	NM_002422.3	Endogenous
**MT1A**	NM_002423.3	Endogenous
**MTFP1**	NM_004994.2	Endogenous
**MTSS1**	NM_002444.2	Endogenous
**MYC**	NM_002447.1	Endogenous
**NANOG**	NM_005946.2	Endogenous
**NAV2**	NM_001003704.2	Endogenous
**NFATC1**	NM_014751.4	Endogenous
**NKD1**	NM_002467.3	Endogenous
**NLK**	NM_024865.2	Endogenous
**NODAL**	NM_001111018.1	Endogenous
**NOTCH1**	NM_172389.1	Endogenous
**NRCAM**	NM_033119.4	Endogenous
**NRP1**	NM_016231.4	Endogenous
**NTRK2**	NM_018055.3	Endogenous
**NUDT13**	NM_017617.3	Endogenous
**OCLN**	NM_005010.4	Endogenous
**PDGFRA**	NM_003873.5	Endogenous
**PDGFRB**	NM_001007097.1	Endogenous
**PITX2**	NM_001283014.1	Endogenous
**PLAUR**	NM_002538.3	Endogenous
**PLEK2**	NM_006206.3	Endogenous
**PORCN**	NM_002609.3	Endogenous
**POU5F1**	NM_000325.5	Endogenous
**PPAP2B**	NM_001005376.1	Endogenous
**PPARD**	NM_016445.1	Endogenous
**PRICKLE1**	NM_022825.2	Endogenous
**PRMT6**	NM_002701.4	Endogenous
**PTCH1**	NM_003713.3	Endogenous
**PTGS2**	NM_006238.4	Endogenous
**PTK2**	NM_153026.1	Endogenous
**PTP4A1**	NM_018137.1	Endogenous
**RAC1**	NM_000264.3	Endogenous
**RGS2**	NM_000963.1	Endogenous
**RHOA**	NM_153831.2	Endogenous
**RUNX2**	NM_003463.3	Endogenous
**RUVBL1**	NM_198829.1	Endogenous
**SERPINE1**	NM_002923.1	Endogenous
**SFRP1**	NM_001664.2	Endogenous
**SFRP2**	NM_001002.3	Endogenous
**SFRP4**	NM_004348.3	Endogenous
**SIX1**	NM_003707.2	Endogenous

**SKP2**	NM_001165413.1	Endogenous
**SMAD2**	NM_003012.3	Endogenous
**SMO**	NM_003013.2	Endogenous
**SNAI1**	NM_003014.2	Endogenous
**SNAI2**	NM_005982.3	Endogenous
**SNAI3**	NM_005983.2	Endogenous
**SOX10**	NM_005901.5	Endogenous
**SOX17**	NM_005631.3	Endogenous
**SOX2**	NM_005985.2	Endogenous
**SOX9**	NM_003068.3	Endogenous
**SPARC**	NM_178310.1	Endogenous
**SPP1**	NM_006941.3	Endogenous
**STAT3**	NM_022454.3	Endogenous
**STEAP1**	NM_003106.2	Endogenous
**TCF3**	NM_000346.2	Endogenous
**TCF4**	NM_003118.2	Endogenous
**TCF7**	NM_000582.2	Endogenous
**TCF7L1**	NM_139276.2	Endogenous
**TCF7L2**	NM_012449.2	Endogenous
**TFPI2**	NM_003200.3	Endogenous
**TGFB1**	NM_001083962.1	Endogenous
**TGFB2**	NM_201633.2	Endogenous
**TGFB3**	NM_031283.1	Endogenous
**TIMP1**	NM_001146274.1	Endogenous
**TLE1**	NM_006528.3	Endogenous
**TMEFF1**	NM_000660.3	Endogenous
**TMEM132A**	NM_003238.2	Endogenous
**TSPAN13**	NM_003239.2	Endogenous
**TWIST1**	NM_003254.2	Endogenous
**VANGL2**	NM_005077.3	Endogenous
**VCAN**	NM_003692.3	Endogenous
**VEGFA**	NM_017870.3	Endogenous
**VIM**	NM_014399.3	Endogenous
**VPS13A**	NM_000474.3	Endogenous
**WIF1**	NM_020335.2	Endogenous
**WISP1**	NM_004385.3	Endogenous
**WISP2**	NM_001025366.1	Endogenous
**WNT1**	NM_003380.2	Endogenous
**WNT10A**	NM_033305.2	Endogenous
**WNT11**	NM_007191.2	Endogenous
**WNT2**	NM_080838.1	Endogenous
**WNT2B**	NM_003881.2	Endogenous
**WNT3**	NM_005430.2	Endogenous
**WNT3A**	NM_025216.2	Endogenous
**WNT4**	NM_004626.2	Endogenous
**WNT5A**	NM_003391.2	Endogenous
**WNT5B**	NM_024494.1	Endogenous
**WNT6**	NM_030753.3	Endogenous
**WNT7A**	NM_033131.2	Endogenous
**WNT7B**	NM_030761.3	Endogenous
**WNT8A**	NM_003392.3	Endogenous
**WNT9A**	NM_032642.2	Endogenous
**ZEB1**	NM_006522.3	Endogenous
**ZEB2**	NM_004625.3	Endogenous

Supplementary Table 2. Ingenuity Pathway Analysis (IPA) Summary on significantly modulated gene expression by FZD4-miR-SNP and WNT5A/B activities in H1299 cells using NanoString Wnt/EMT signaling pathway gene expression profiling.

INGENUITY
PATHWAY ANALYSIS

Analysis Name: result-p0.075-WNT5A-WTvsWNT5A-SNP-A549 - 2017-01-20 04:18 PM
Analysis Creation Date: 2017-07-12
Build version: 448560M
Content version: 31813283 (Release Date: 2016-12-05)

Analysis Settings

Reference set: Ingenuity Knowledge Base (Genes Only)
Relationship to include: Direct and Indirect
Includes Endogenous Chemicals
Optional Analyses: My Pathways My List

Filter Summary:
Consider only molecules and/or relationships where
(species = Human) AND
(confidence = Experimentally Observed)

Top Canonical Pathways

Name	p-value	Overlap	
Wnt/-catenin Signaling	2.19E-16	7.7 %	13/169
Role of Macrophages, Fibroblasts and Endothelial Cells in Rheumatoid Arthritis	2.20E-14	4.5 %	14/309
Colorectal Cancer Metastasis Signaling	3.09E-14	5.3 %	13/247
Regulation of the Epithelial-Mesenchymal Transition Pathway	3.58E-14	6.3 %	12/189
Pancreatic Adenocarcinoma Signaling	1.40E-11	7.6 %	9/118

Top Upstream Regulators

Upstream Regulator	p-value of overlap	Predicted Activation
NOTCH1	4.00E-26	Inhibited
ERBB2	1.57E-25	Inhibited
STAT3	7.60E-25	Inhibited
CTNNB1	8.27E-25	Inhibited
HRAS	6.46E-24	Inhibited

Top Diseases and Bio Functions

Diseases and Disorders

Name	p-value	#Molecules
Cancer	9.05E-10 - 1.43E-29	50
Organismal Injury and Abnormalities	9.05E-10 - 1.43E-29	51
Developmental Disorder	7.25E-10 - 2.45E-25	36
Tumor Morphology	8.71E-10 - 9.61E-25	37
Gastrointestinal Disease	3.57E-10 - 6.18E-18	48

Molecular and Cellular Functions

Cellular Movement	7.94E-10 - 7.66E-29	42
Cell Death and Survival	9.05E-10 - 2.04E-25	43
Cellular Development	9.11E-10 - 4.73E-25	47
Cellular Growth and Proliferation	9.11E-10 - 4.73E-25	49
Gene Expression	4.44E-11 - 2.54E-19	36

Name	p-value	#Molecules
Cardiac Hypertrophy	2.19E-01 - 4.22E-11	13
Cardiac Fibrosis	6.56E-02 - 1.29E-10	10
Cardiac Arrythmia	2.97E-02 - 1.23E-09	10
Congenital Heart Anomaly	1.16E-01 - 2.23E-08	9
Cardiac Inflammation	1.02E-01 - 4.45E-06	5

Hepatotoxicity

Name	p-value	#Molecules
Hepatocellular Carcinoma	1.34E-01 - 2.25E-14	20
Liver Hyperplasia/Hyperproliferation	1.34E-01 - 2.25E-14	31
Liver Proliferation	3.46E-02 - 1.35E-10	10
Liver Cirrhosis	1.00E-02 - 2.71E-10	10
Liver Necrosis/Cell Death	5.14E-02 - 3.26E-09	9

Nephrotoxicity

Name	p-value	#Molecules
Renal Proliferation	1.74E-02 - 1.12E-10	10
Renal Damage	2.09E-01 - 2.35E-09	8
Renal Necrosis/Cell Death	2.48E-02 - 1.26E-08	12
Glomerular Injury	6.56E-02 - 1.16E-07	11
Renal Hypertrophy	1.74E-02 - 3.02E-06	4

Physiological System Development and Function

Name	p-value	#Molecules
Cardiovascular System Development and Function	5.35E-10 - 5.15E-27	38
Organismal Development	9.11E-10 - 5.15E-27	45
Embryonic Development	9.11E-10 - 2.45E-25	41
Tissue Morphology	9.05E-10 - 2.45E-25	40
Connective Tissue Development and Function	6.84E-10 - 5.96E-25	38

Top Tox Functions

Assays: Clinical Chemistry and Hematology

Name	p-value	#Molecules
Increased Levels of Alkaline Phosphatase	2.51E-03 - 5.39E-10	8
Decreased Levels of Albumin	5.01E-03 - 3.69E-05	2
Increased Levels of Creatinine	1.38E-04 - 1.38E-04	3
Increased Levels of Albumin	2.51E-03 - 1.71E-04	4
Increased Levels of CRP	5.01E-03 - 5.01E-03	1

Cardiotoxicity

Top Networks

ID	Associated Network Functions	Score
1	Cell Cycle, Gene Expression, Cellular Development	22
2	Cellular Development, Cellular Growth and Proliferation, Organ Development	15
3	Organismal Injury and Abnormalities, Cellular Movement, Nervous System Development and Function	15
4	Cellular Development, Reproductive System Development and Function, Embryonic Development	13
5	Connective Tissue Development and Function, Connective Tissue Disorders, Organ Morphology	10

Top Tox Lists

Name	p-value	Overlap	
Increases Renal Proliferation	2.57E-12	6.9 %	10/145
Increases Glomerular Injury	5.46E-11	9.0 %	8/89
Cardiac Hypertrophy	6.77E-11	2.9 %	13/454
Cardiac Fibrosis	6.97E-11	5.0 %	10/202
Liver Proliferation	2.49E-10	4.3 %	10/230

Top Analysis-Ready Molecules

Expr Fold Change up-regulated

Molecules	Expr. Value	Expr. Chart
**CSNK1A1**	↑ 2.161	
**WNT5A**	↑ 1.336	
**ERBB3**	↑ 1.173	
**TSPAN13**	↑ 1.113	
**CALM1 (includes others)**	↑ 1.103	
**FN1**	↑ 1.092	
**CEBPD**	↑ 1.078	
**HPRT1**	↑ 1.070	
**DKK1**	↑ 1.068	

Expr Fold Change down-regulated

Molecules	Expr. Value	Expr. Chart
**IL6**	↓ -2.411	
**CDH2**	↓ -1.628	
**PTGS2**	↓ -1.580	
**NAV2**	↓ -1.408	
**BMP2**	↓ -1.349	
**FOSL1**	↓ -1.327	
**MYC**	↓ -1.316	
**AXIN1**	↓ -1.308	
**CTGF**	↓ -1.291	
**PLPP3**	↓ -1.278	

Supplementary Table 3. Ingenuity Pathway Analysis (IPA) Summary on significantly modulated gene expression by co-expression of miR-204 and FZD4-miR-SNP in H1299 cells on NanoString Wnt/E M T signaling pathway gene expression profiling.

INGENUITY
PATHWAY ANALYSIS

Analysis Name: result-p0.075-SNP-miR204-wnt5avsSNP-miRcon-wnt5a-H1299mir - 2017-01-20 03:42 PM
Analysis Creation Date: 2017-07-12
Build version: 448560M
Content version: 31813283 (Release Date: 2016-12-05)

Analysis Settings

Reference set: Ingenuity Knowledge Base (Genes Only)
Relationship to include: Direct and Indirect
Includes Endogenous Chemicals
Optional Analyses: My Pathways My List

Filter Summary:
Consider only molecules and/or relationships where
(species = Human) AND
(confidence = Experimentally Observed)

Top Canonical Pathways

Name	p-value	Overlap	
Wnt/-catenin Signaling	6.26E-21	7.7 %	13/169
Role of Macrophages, Fibroblasts and Endothelial Cells in Rheumatoid Arthritis	2.62E-12	3.2 %	10/309
Role of Osteoblasts, Osteoclasts and Chondrocytes in Rheumatoid Arthritis	7.85E-12	3.9 %	9/232
Colorectal Cancer Metastasis Signaling	1.38E-11	3.6 %	9/247
Molecular Mechanisms of Cancer	1.73E-11	2.7 %	10/374

Top Upstream Regulators

Upstream Regulator	p-value of overlap	Predicted Activation
MBD3	6.77E-14	
CYR61	1.21E-12	
TNF	2.86E-09	
estrogen receptor	1.40E-08	
WNT3A	2.16E-08	

Top Diseases and Bio Functions

Diseases and Disorders

Name	p-value	#Molecules
Gastrointestinal Disease	1.28E-03 - 2.80E-09	23
Hepatic System Disease	1.05E-03 - 2.80E-09	11
Inflammatory Disease	9.79E-04 - 2.80E-09	9
Organismal Injury and Abnormalities	1.28E-03 - 2.80E-09	25
Connective Tissue Disorders	1.28E-03 - 2.70E-08	17

Molecular and Cellular Functions

Name	p-value	#Molecules
Cellular Growth and Proliferation	1.28E-03 - 2.26E-12	23
Cellular Movement	1.28E-03 - 7.11E-11	19
Cellular Development	1.28E-03 - 3.04E-10	22
Cell Death and Survival	1.28E-03 - 3.39E-10	21
Gene Expression	1.02E-03 - 4.18E-09	17

Physiological System Development and Function

Name	p-value	#Molecules
Tissue Development	1.28E-03 - 2.26E-12	23
Embryonic Development	1.28E-03 - 2.86E-12	21
Organismal Development	1.28E-03 - 2.86E-12	24
Connective Tissue Development and Function	1.28E-03 - 3.04E-10	19
Organ Development	1.28E-03 - 1.07E-09	19

Top Tox Functions

Assays: Clinical Chemistry and Hematology

Name	p-value	#Molecules
Increased Levels of Hematocrit	6.53E-03 - 6.53E-03	2
Increased Levels of Alkaline Phosphatase	8.81E-02 - 8.81E-02	1
Increased Levels of Red Blood Cells	1.23E-01 - 1.23E-01	1

Cardiotoxicity

Name	p-value	#Molecules
Congenital Heart Anomaly	4.26E-02 - 1.28E-04	3
Cardiac Arteriopathy	1.24E-02 - 1.28E-03	3
Cardiac Damage	3.83E-03 - 3.83E-03	1
Cardiac Fibrosis	2.53E-02 - 6.37E-03	1
Cardiac Necrosis/Cell Death	8.91E-03 - 8.91E-03	1

Hepatotoxicity

Name	p-value	#Molecules
Liver Cirrhosis	2.80E-09 - 2.80E-09	6
Hepatocellular Carcinoma	7.04E-02 - 8.74E-04	6
Liver Hyperplasia/Hyperproliferation	8.69E-02 - 8.74E-04	14
Liver Fibrosis	5.96E-02 - 1.05E-03	4
Liver Dysplasia	7.64E-03 - 7.64E-03	1

Nephrotoxicity

Name	p-value	#Molecules
Renal Damage	1.90E-02 - 1.90E-02	1
Renal Tubule Injury	1.90E-02 - 1.90E-02	1
Glomerular Injury	1.33E-01 - 2.78E-02	1
Renal Hydronephrosis	1.11E-01 - 1.11E-01	1
Kidney Failure	2.09E-01 - 2.09E-01	1

Top Networks

ID	Associated Network Functions	Score
1	Embryonic Development, Organismal Development, Cellular Development	41
2	Cancer, Endocrine System Disorders, Organismal Injury and Abnormalities	12
3	Cell-To-Cell Signaling and Interaction, Hematological System Development and Function, Immune Cell Trafficking	10

Top Tox Lists

Name	p-value	Overlap
Cell Cycle: G1/S Checkpoint Regulation	8.07E-05	4.5 % 3/66
Primary Glomerulonephritis Biomarker Panel (Human)	8.59E-05	18.2 % 2/11
TGF- Signaling	5.88E-03	2.2 % 2/90
Aryl Hydrocarbon Receptor Signaling	1.75E-02	1.3 % 2/159
Recovery from Ischemic Acute Renal Failure (Rat)	1.78E-02	7.1 % 1/14

Top Analysis-Ready Molecules

Expr Fold Change up-regulated

Molecules	Expr. Value	Expr. Chart
**WNT2**	↑ 2.062	
**JAG1**	↑ 1.304	
**BOD1**	↑ 1.244	
**STEAP1**	↑ 1.227	
**DAAM1**	↑ 1.207	
**MAP1B**	↑ 1.192	
**SERPINE1**	↑ 1.190	
**RUVBL1**	↑ 1.143	
**MTFP1**	↑ 1.124	
**WNT7B**	↑ 1.076	

Expr Fold Change down-regulated

Molecules	Expr. Value	Expr. Chart
**WNT7A**	↓ -3.500	
**WNT5A**	↓ -2.534	
**FRZB**	↓ -2.335	
**LRP1**	↓ -1.396	
**ITGA5**	↓ -1.321	
**F11R**	↓ -1.231	
**BTRC**	↓ -1.209	
**DAB2**	↓ -1.208	
**CTNNBIP1**	↓ -1.207	
**TGFB2**	↓ -1.157	

Supplementary Table 4. Top-scored network associated with FZD4-miR-SNP and WNT5A/B Activities as well as ectopic expression of miR-204 in NSCLC H1299 cells by quantitative NanoString Wnt/EMT signaling pathway gene expression profiling and Ingenuity Pathway Analysis (IPA).

I. Top Scored Network Associated with FZD4-miR-SNP and WNT5A/B Activities in NSCLC H1299 Cells

ID	Molecules in Network	Score	Focus Molecules	Top Diseases and Functions
1	Ap1,CALM1 (includes others),caspase,CCND1,CD3,chemokine,Ck2,Endothelin,ETS2,FN1,Focal adhesion kinase,G protein alphai,Gsk3,Histone h3,HPRT1,ID2,IgG2b,IL6,Mapk,Metalloprotease,Mmp,MTORC1,MYC,NRP1,P38 MAPK,Pdgfr,Pkc(s),Rac,RAC1,Ras,Ras homolog,RNA polymerase II,Secretase gamma,TGFB1,Vegf	22	11	Cell Cycle, Gene Expression, Cellular Development
2	Alp,Alpha actin,Alpha tubulin,AMPK,Atrial Natriuretic Peptide,BMP2,C/ebp,CEBPD,Collagen type ix,Collagen(s),Cpla2,DKK1,ERK,Growth hormone,HDL,Ifn,IL1,ILK,KLF5,Laminin1,LDL,Myosin,NADPH oxidase,Nos,NOTCH1,PARP,Pdgf (complex),PI3K (family),PRKAA,Pro-inflammatory Cytokine,PTGS2,Rock,SAA,SERPINE1,Tnf (family)	15	8	Cellular Development, Cellular Growth and Proliferation, Organ Development
3	26s Proteasome,48s,Actin,ADCY,Alpha catenin,Cadherin,Calmodulin,Cg,Creb,ERBB3,F Actin,FSH,GJA1,Hedgehog,Hsp27,Hsp70,Hsp90,Insulin,Lh,MAP1B,MIRLET7,NKD1,p85 (pik3r),PI3K (complex),Pka,Pka catalytic subunit,PLC,Proinsulin,PTP4A1,PTPase,SRC (family),STEAP1,TSPAN13,tubulin (complex),VEGFA	15	8	Organismal Injury and Abnormalities, Cellular Movement, Nervous System Development and Function
4	7S NGF,AHR,Alpha 1 antitrypsin,Angiotensin II receptor type 1,Caveolin,Cdc2,Cdk,Ctbp,Cyclin A,Cyclin D,Cyclin E,E2f,estrogen receptor,Hdac,HISTONE,histone deacetylase,Histone h4,JAG1,JUN/JUNB/JUND,KRT7,N-cor,NFkB (complex),Notch,P glycoprotein,Rb,Rxr,sphingomyelinase,TCF,TFPI2,thymidine kinase,TLE1,trypsin,TWIST,TWIST1,ZEB1	13	7	Cellular Development, Reproductive System Development and Function, Embryonic Development
5	Adaptor protein 1,Bcl9-Cbp/p300-Ctnnb1-Lef/Tcf,Betacatenin/TCF,BMP,Cbp/p300,collagen,Collagen Alpha1,Collagen type II,CTGF,Ecm,elastase,Eotaxin,ERK1/2,Fibrin,Igf,Integrin,Integrin alpha 6 beta 1,Laminin,MMP7,PLPP3,Smad,SMAD1/5,Smad1/5/8,Smad2/3,Smad2/3-Smad4,SMOOTH MUSCLE ACTIN,Stat3-Stat3,TCF/LEF,Tenascin,Tgf beta,TGFB2,TGFBR,TMSB4,WISP2,WNT5A	10	6	Connective Tissue Development and Function, Connective Tissue Disorders, Organ Morphology
6	APC (complex),APC/APC2,AXIN1,BCR (complex),Cofilin,CSNK1A1,Dishevelled,EGFR,FOSL1,Frizzled,Gm-csf,GOT,Hspg,IGFBP4,IgG2a,IL23,JAK,LRP,MAP2K1/2,Mek,Mlc,Mucin,NRG (family),p70 S6k,Pak,PDGF BB,PI3K p85,PP2A,Ppp2c,Raf,Rsk,Sos,STAT5a/b,transglutaminase,VAV	8	5	Digestive System Development and Function, Organ Morphology, Organismal Development
7	Akt,c-Src,calpain,CDH2,Ciap,Collagen type I,Collagen type III,Collagen type IV,Collagen type V,Fascin,Fgf,Fgfr,Fibrinogen,FKHR,Integrin alpha 2 beta 1,Integrin alpha 3 beta 1,Integrin alpha 4 beta 1,Integrin alpha 5 beta 1,ITGA5,ITGB1,JINK1/2,Lfa-1,MTORC2,N-Cadherin,NFkB (family),NfkB1-RelA,Pdgf Ab,PLC gamma,Ptk,Rap1,SYK/ZAP,Talin,Vegf Receptor,Vla-4,Wnt	4	3	Cell-To-Cell Signaling and Interaction, Embryonic Development, Organ Development
8	B2M,Calcineurin protein(s),cytochrome C,DSP,Fc gamma receptor,Fcer1,hemoglobin,IFN Beta,Ifn gamma,Iga,Ige,IgG,IgG1,Igg3,Igm,Ikb,IKK (complex),IL12 (complex),IL12 (family),Immunoglobulin,Interferon alpha,Jnk,Ldh (complex),MHC Class I (complex),MHC CLASS I (family),MHC Class II (complex),NFAT (complex),Nfat (family),STAT3,TCR,TEAD,Tlr,Tnf receptor,TSH,tyrosine kinase	4	3	Increased Levels of Albumin, Cancer, Hematological Disease
9	10-nitrooleate,ADAP1,ADGRE1,apyrase,ARHGAP27,ARHGEF19,BPI,Ca2+,CCL24,CCL3L1,Cyba-Ncf1c-Ncf2-Nox-Ncf4,CYP4F3,dehydroascorbic acid,GCA,Gm-Csf Receptor,GNRH,GTPase,GYPC,ICAM1,ICAM3,IL17B,IL36RN,NAV2,NFkB (complex),Pik3r,Pkc(s),RAC1,ribose,RUVBL1,SENP6,SH3BP1,Shc,STAT,tretinoin,Ubiquitin	4	3	Cell-To-Cell Signaling and Interaction, Hair and Skin Development and Function, Cellular Movement

II. Top Scored Network Associated Ectopic Expression of miR-204, FZD4-miR-SNP, and WNT5A/B in H1299 Cells

ID	Molecules in Network	Score	Focus Molecules	Top Diseases and Functions
1	Alp,APC,atypical protein kinase C,Collagen type I,CTNNBIP1,DAAM1,DAB2,Dishevelled,DVL2,ERK1/2,F11R,Frizzled,FRZB,Gsk3,GTPase,Hedgehog,Integrin,JAG1,Laminin,LRP,LRP1,LRP6,PDGF BB,Rock,Smad,SOX9,TCF,Tgf beta,TGFB2,TGFBR,Wnt,WNT2,WNT5A,WNT7A,WNT7B	41	16	Embryonic Development, Organismal Development, Cellular Development
2	26s Proteasome,ACTB,Androgen-AR,APP,AQP5,BOD1,BTRC,CAP2,CCDC50,DPCD,EFHD2,EGFR,Histone h3,Histone h4,HOXA11,Insulin,KLHL17,Lrrfip2,MTFP1,MTORC1,NFkB (complex),PCYT1B,PROSER2,REG1A,RNA polymerase II,RUFY3,RUVBL1,SETBP1,SPANXN3,TCP11L2,TPST1,TXNL1,UBC,VPS13A,VSNL1	12	6	Cancer, Endocrine System Disorders, Organismal Injury and Abnormalities
3	Actin,Akt,Ap1,calpain,CaMKII,CCND1,Cg,Collagen(s),Creb,E2f,ERK,estrogen receptor,Fibrinogen,Focal adhesion kinase,FSH,IL1,ITGA5,Jnk,LDL,Lh,MAP1B,Mapk,Mek,Nfat (family),P38 MAPK,Pdgf (complex),PI3K (complex),Pka,Pkc(s),Rac,Ras,SERPINE1,Smad2/3,STEAP1,Tnf (family)	10	5	Cell-To-Cell Signaling and Interaction, Hematological System Development and Function, Immune Cell Trafficking

Supplementary Table 5. Molecules associated with FZD4-miR-SNP and WNT5A/B Activities as well as ectopic expression of miR-204 in Wnt/b-catenin signaling pathways in NSCLC H1299 cells by quantitative NanoString Wnt/EMT signaling pathway gene expression profiling and Ingenuity Pathway Analysis (IPA).

Gene Symbol	Entrez Gene Name	Expr p-value	Expr False Discovery Rate (q-value)	Expr Fold Change	Expected	subcellular Location	Type(s)
**I. Molecules associated with FZD4-miR-SNP and WNT5A/B activities in Wnt/b-Catenin signaling pathways**							
AXIN1	axin 1	0.0315	0.237	-1.308	Down	Cytoplasm	other
CCND1	cyclin D1	0.0147	0.182	-1.054	Up	Nucleus	transcription regulator
CDH2	cadherin 2	0.0168	0.182	-1.628		Plasma Membrane	other
CSNK1A1	casein kinase 1 alpha 1	0.0548	0.283	2.161	Up	Cytoplasm	kinase
DKK1	dickkopf WNT signaling pathway inhibitor 1	0.0439	0.247	1.068	Down	Extracellular Space	growth factor
GJA1	gap junction protein alpha 1	0.0101	0.182	-1.151	Up	Plasma Membrane	transporter
ILK	integrin linked kinase	0.0599	0.287	-1.069		Plasma Membrane	kinase
MMP7	matrix metallopeptidase 7	0.0428	0.247	-1.06	Up	Extracellular Space	peptidase
MYC	MYC proto-oncogene	0.0128	0.182	-1.316	Up	Nucleus	transcription regulator
TGFB1	transforming growth factor beta 1	0.0738	0.313	-1.098	Down	Extracellular Space	growth factor
TGFB2	transforming growth factor beta 2	0.00359	0.135	-1.136	Down	Extracellular Space	growth factor
TLE1	transducin like enhancer of split 1	0.0179	0.182	-1.197		Nucleus	transcription regulator
WNT5A	Wnt family member 5A	0.00993	0.182	1.336	Up	Extracellular Space	cytokine
**II. Molecules associated with co-expression of miR-204 and FZD4-miR-SNP in Wnt/b-Catenin signaling pathways**							
APC	APC, WNT signaling pathway regulator	0.0249	0.398	-1.122	Down	Nucleus	enzyme
BTRC	beta-transducin repeat containing E3 ubiquitin protein ligase	0.0637	0.493	-1.209	Up	Cytoplasm	enzyme
CCND1	cyclin D1	0.00449	0.222	-1.151	Up	Nucleus	transcription regulator
DVL2	dishevelled segment polarity protein 2	0.0546	0.493	-1.127	Up	Cytoplasm	other
FRZB	frizzled-related protein	0.0589	0.493	-2.335	Down	Extracellular Space	other
LRP1	LDL receptor related protein 1	0.0725	0.493	-1.396	Up	Plasma Membrane	transmembrane receptor
LRP6	LDL receptor related protein 6	0.0458	0.493	-1.068	Up	Plasma Membrane	transmembrane receptor
SOX9	SRY-box 9	0.0656	0.493	-1.02	Down	Nucleus	transcription regulator
TGFB2	transforming growth factor beta 2	0.00518	0.222	-1.157	Down	Extracellular Space	growth factor
WNT2	Wnt family member 2	0.016	0.276	2.062	Up	Extracellular Space	cytokine
WNT5A	Wnt family member 5A	0.0017	0.222	-2.534	Up	Extracellular Space	cytokine
WNT7A	Wnt family member 7A	0.0538	0.493	-3.5	Up	Extracellular Space	cytokine
WNT7B	Wnt family member 7B	0.0708	0.493	1.076	Up	Extracellular Space	other

Supplementary Figures

Figure S1, Full Length Western-blot images for Fig. 1E. A and **B** show images for anti-Flag with different intensities and **C** for anti-β-Actin from the same Western-blot. Images were obtained by a LI-COR Odyssey Imaging system with equipped imaging analysis software (LI-COR Biotechnology, Lincoln, NE).

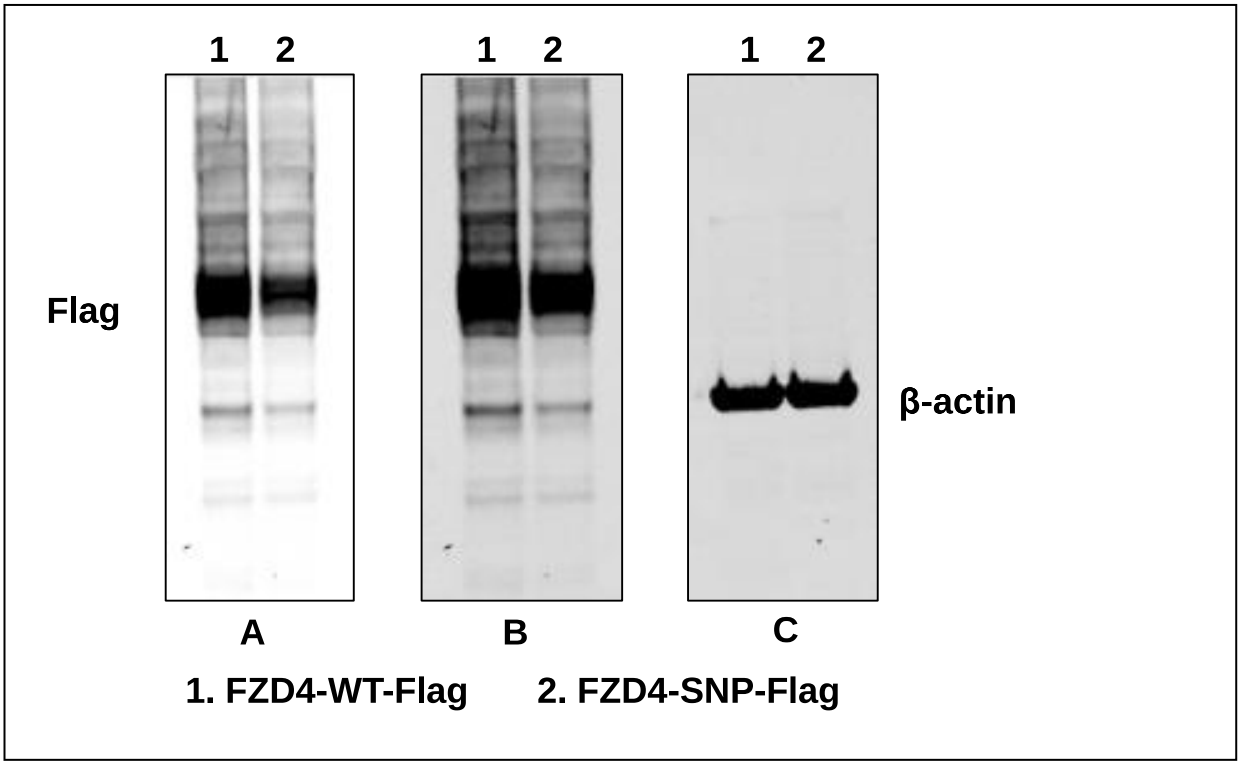

Figure S2, Full Length gel images for Fig. 2C.

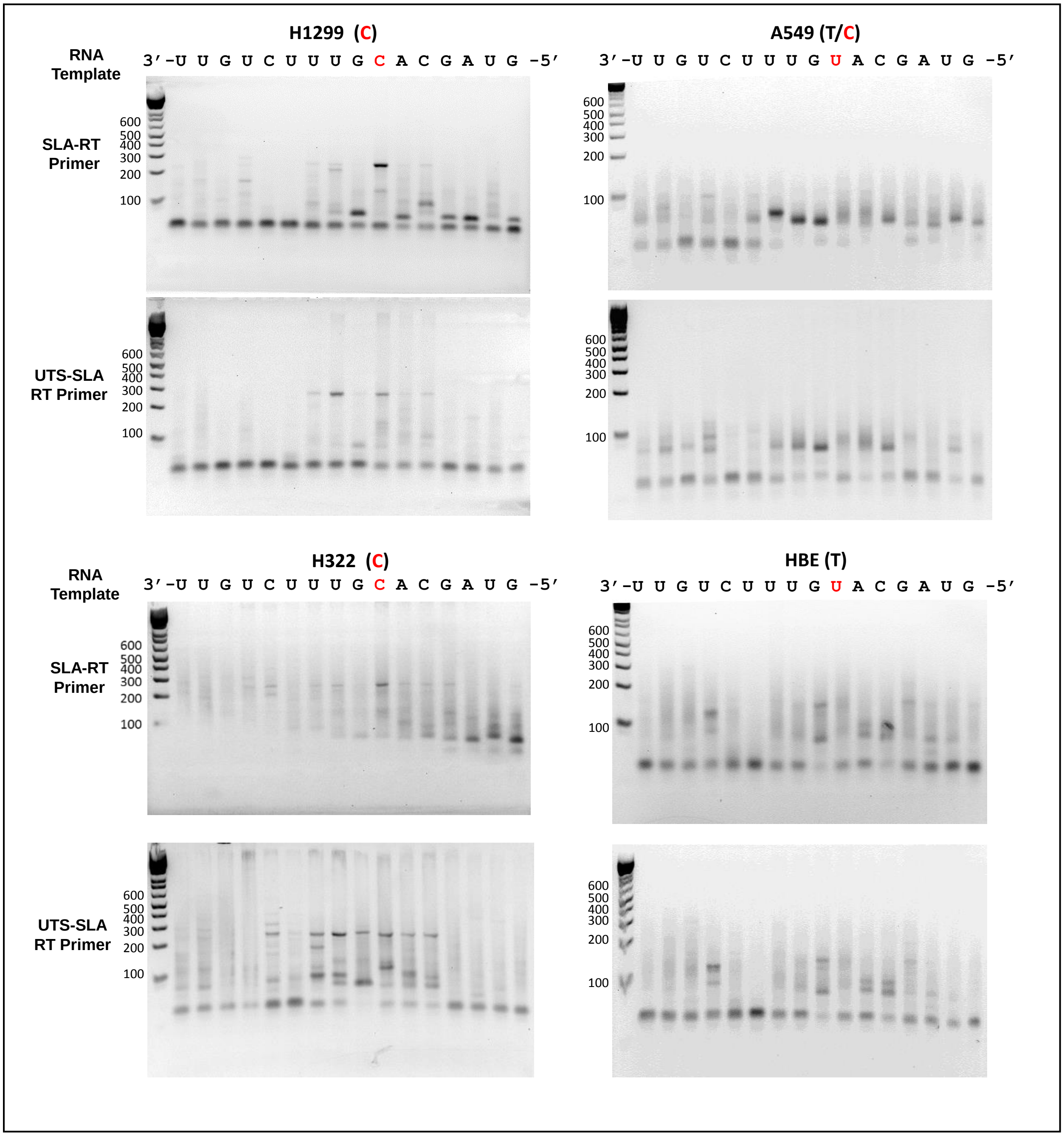

Figure S3, Full Length individual gel images for Fig. 2D. The areas and locations that were cropped for Figure 2D are indicated in the boxes.

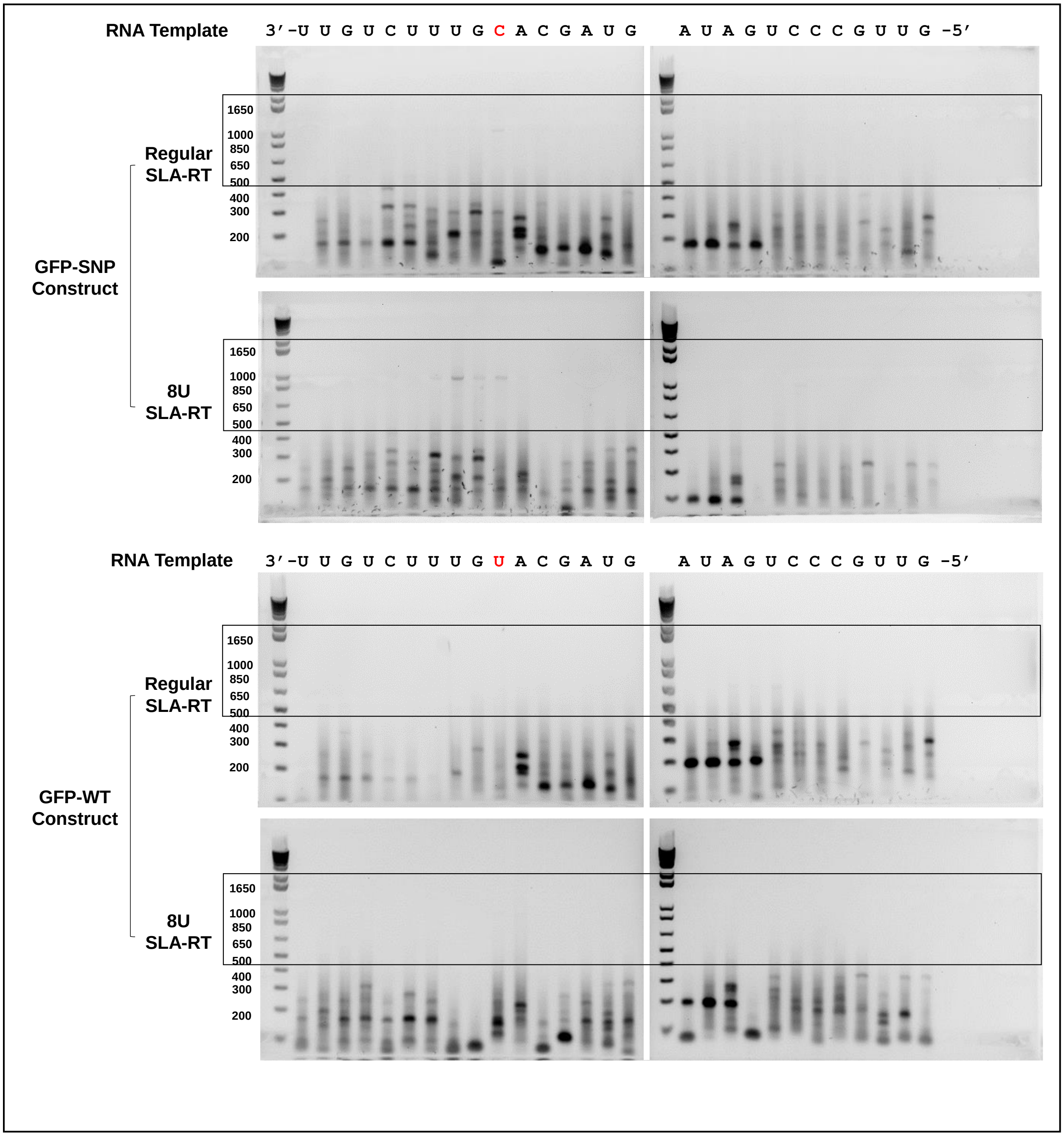

Figure S4. Full length Western-blot images for Fig. 3C

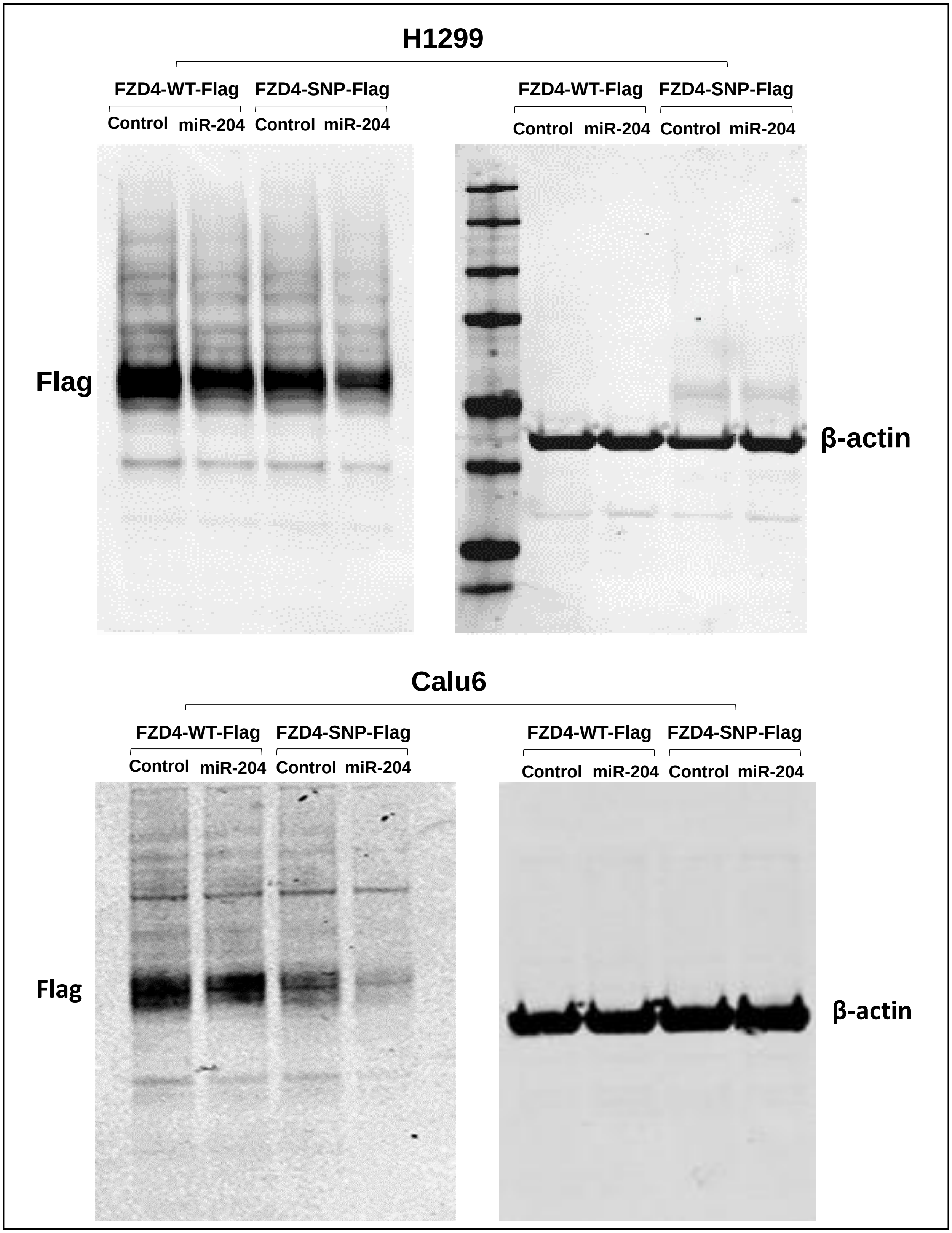

Figure S5. Expression profiling of miR-204 in normal human bronchial epithelial cell (HBEC) and non-small cell lung cancer cell (NSCLC) lines by miRNA microarray analysis **(A)** and association of FZD4 and miR-204 expression in primary NSCLC adenocarcinoma (LUAD) and squamous carcinoma (LUSC) from The Cancer Genome Atlas (TCGA) (**https://cancergenome.nih.gov**) database **(B).** A reciprocal correlation between miR-204 and FZD4 gene expression were shown in LUAD and LUSC tumors from TCGA data portal.

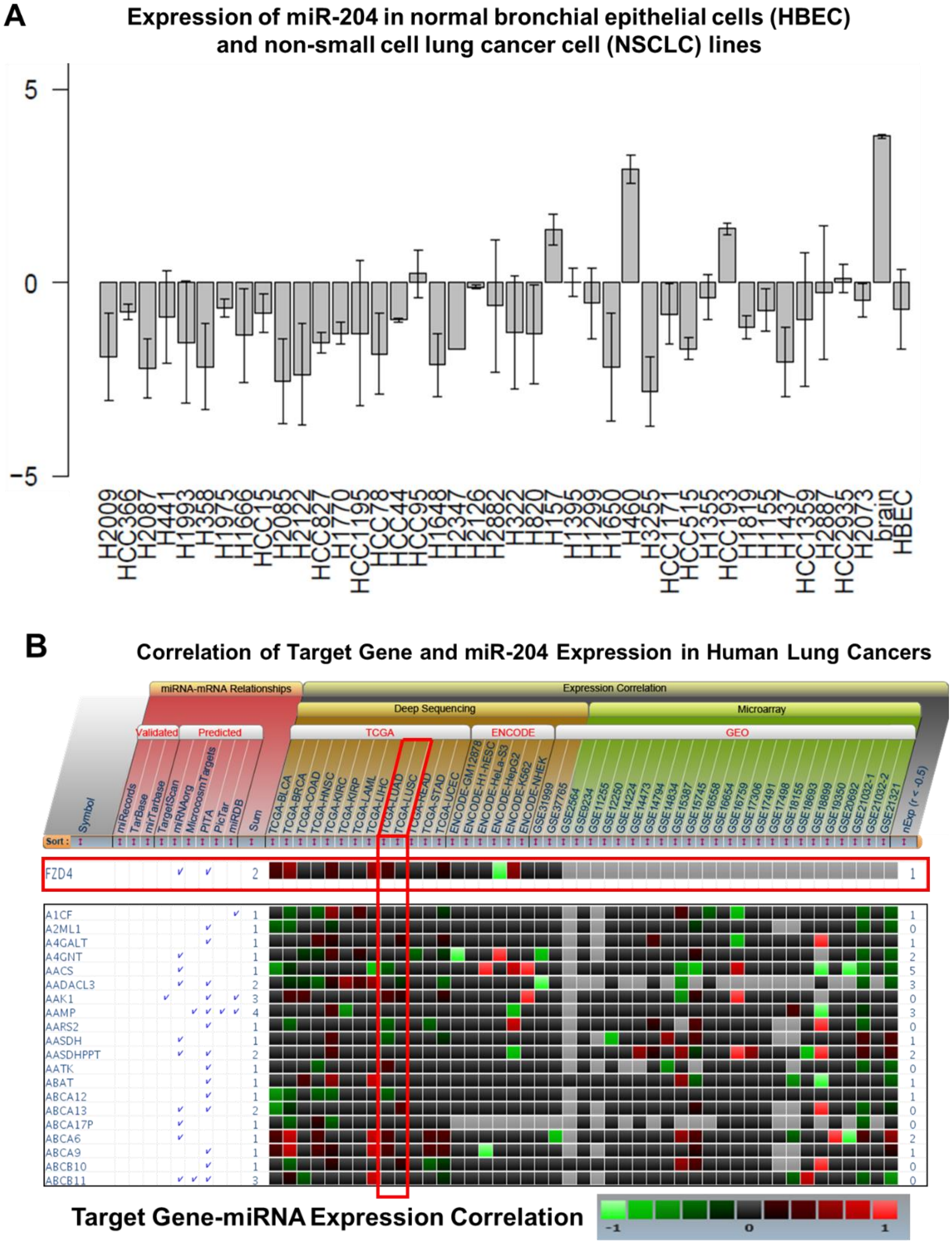

Supplement: Supplementary file 1 — Supplementary Information [file 41598_2017_9604_MOESM1_ESM.pdf]
